# Selective IL-1 activity on CD8^+^ T cells empowers antitumor immunity and synergizes with neovasculature-targeted TNF for full tumor eradication

**DOI:** 10.1136/jitc-2021-003293

**Published:** 2021-11-12

**Authors:** Bram Van Den Eeckhout, Leander Huyghe, Sandra Van Lint, Elianne Burg, Stéphane Plaisance, Frank Peelman, Anje Cauwels, Gilles Uzé, Niko Kley, Sarah Gerlo, Jan Tavernier

**Affiliations:** 1 VIB-UGent Center for Medical Biotechnology, Ghent, Belgium; 2 Department of Biomolecular Medicine, Ghent University, Ghent, Belgium; 3 VIB Nucleomics Core, Leuven, Belgium; 4 IRMB, University Montpellier, INSERM, CNRS, Montpellier, France; 5 Orionis Biosciences Inc, Waltham, Massachusetts, USA

**Keywords:** cytokines, CD8-positive t-lymphocytes, neovasularization, pathologic, adjuvants, immunologic, vaccination

## Abstract

**Background:**

Clinical success of therapeutic cancer vaccines depends on the ability to mount strong and durable antitumor T cell responses. To achieve this, potent cellular adjuvants are highly needed. Interleukin-1β (IL-1β) acts on CD8^+^ T cells and promotes their expansion and effector differentiation, but toxicity and undesired tumor-promoting side effects hamper efficient clinical application of this cytokine.

**Methods:**

This ‘cytokine problem’ can be solved by use of AcTakines (Activity-on-Target cytokines), which represent fusions between low-activity cytokine mutants and cell type-specific single-domain antibodies. AcTakines deliver cytokine activity to *a priori* selected cell types and as such evade toxicity and unwanted off-target side effects. Here, we employ subcutaneous melanoma and lung carcinoma models to evaluate the antitumor effects of AcTakines.

**Results:**

In this work, we use an IL-1β-based AcTakine to drive proliferation and effector functionality of antitumor CD8^+^ T cells without inducing measurable toxicity. AcTakine treatment enhances diversity of the T cell receptor repertoire and empowers adoptive T cell transfer. Combination treatment with a neovasculature-targeted tumor necrosis factor (TNF) AcTakine mediates full tumor eradication and establishes immunological memory that protects against secondary tumor challenge. Interferon-γ was found to empower this AcTakine synergy by sensitizing the tumor microenvironment to TNF.

**Conclusions:**

Our data illustrate that anticancer cellular immunity can be safely promoted with an IL-1β-based AcTakine, which synergizes with other immunotherapies for efficient tumor destruction.

## Background

CD8^+^ T cells are pivotal players in anticancer immunity.^1^ Many cancer immunotherapies, therefore, focus on revitalizing CD8^+^ T cell responses, for example, by interfering with pathways of T cell inhibition.^2^ Unfortunately, immune checkpoint inhibitors are only effective in subsets of patients and additional interventions are required when responsiveness is limited or absent.^3^ Moreover, initially responsive patients will often develop acquired resistance to therapy.^4^ Checkpoint inhibitors are currently evaluated in combination with other strategies, including cancer vaccination and adoptive T cell transfer (ATCT).^5–11^ Yet, the efficacy of these strategies remains relatively low and they could benefit from codelivery of so-called ‘cellular’ adjuvants that improve the expansion, function and peripheral survival of both endogenously generated and adoptively transferred CD8^+^ T cells.^12^

A promising candidate for a cellular adjuvant is the proinflammatory cytokine interleukin-1β (IL-1β),^13^ which empowers proliferation, effector functionality and memory differentiation of CD8^+^ T cells in different experimental models.^14–17^ Recently, Lee *et al.* demonstrated that repeated delivery of wild-type (WT) IL-1β in mice with established B16 melanomas improves the antitumor effect of ATCT by enhancing the formation of tumor-associated antigen (TAA)-specific effector CD8^+^ T cells and their peripheral trafficking and survival.^18^ Despite these promising results, two major hurdles hamper clinical translation of IL-1 activity. First, systemic delivery of WT IL-1β induces damaging toxicities, with maximum tolerated doses calculated from phase I clinical trials ranging from 0.07 to 0.3 µg/kg body weight. This toxicity is dose-dependent and includes influenza-like symptoms at lower doses and cardiovascular issues upon administration of higher amounts of WT IL-1β.^19^ Second, chronic inflammation in the tumor microenvironment (TME) can promote cancer progression and as a major inflammatory mediator, the protumorigenic properties of IL-1β are increasingly recognized.^20^ IL-1β fuels tumor development by a plethora of different mechanisms, including promotion of angiogenesis, metastasis and formation of an immunosuppressive TME, characterized by accumulation of CD11b^+^Gr-1^+^ myeloid-derived suppressor cells and CD11b^+^Ly6G^+^ neutrophils that can both dampen CD8^+^ T cell immunity.^21–24^

Importantly, these limitations stem from IL-1β’s pleiotropic mode of action.^13^ Selective delivery of IL-1β activity to CD8^+^ T cells might limit toxicity and undesired side effects, hence allowing application as a cellular adjuvant in cancer immunotherapy. Selective cytokine activity can be achieved using AcTakines (‘Activity-on-Target cytokines’), which are fusion proteins comprizing a cytokine mutant with reduced receptor affinity and a single-domain antibody (sdAb) directed towards a cell type-specific surface molecule. AcTakines remain inactive *en route* through the body and regain full activity on target cell binding.^25^ Previously, we reported on the development of an AcTakine that targets activity of the IL-1β Q148G mutant to CD8^+^ T cells (CD8α AcTaleukin-1/ALN-1) in an influenza A model.^26^

Here, we demonstrate that CD8α ALN-1 drives antitumor CD8^+^ T cell responses in subcutaneous (s.c.) melanoma (B16) and Lewis lung carcinoma (LLC) models. The potent cellular adjuvant effect of CD8α ALN-1 empowers epitope spreading, expansion and effector functionality of endogenous host T cells and enhances the efficacy of ATCT. CD8α ALN-1 synergizes with a neovasculature-targeted tumor necrosis factor (TNF) AcTakine, leading to complete tumor destruction and formation of immunological memory that protects against secondary tumor challenge. Hence, selective IL-1β activity on CD8^+^ T cells provides a safe way to promote a diverse antitumor T cell response and synergy with other immunotherapies.

## Methods

### Tumor models

Mice were inoculated s.c. in the shaved back with 0.5×10^6^ tumor cells in 50 µL of PBS (14 190–169, Thermo Fisher Scientific) under mild isoflurane anesthesia (Abbott Animal Health). Mice were sacrificed when both perpendicular tumor sides exceeded 10 mm or when 20% reduction in starting body weight was observed.

### Flow cytometry and antibodies

Tissues were collected in PBS and mechanically mashed on 70 µm nylon strainers. Spleens and tumors were treated with a home-made red blood cell lysis buffer (155 mM Na_4_Cl, 12 mM NaHCO_3_ and 127 µM EDTA in PBS *p*H 7.4). Single cell suspensions were incubated for 30 min at 4°C in PBS with anti-mouse CD16/32 to block Fc receptors. Staining was performed for 1 hour at 4°C with antibodies or dye diluted in PBS (see online supplemental table 1). Sample fixation and permeabilization was performed using the Foxp3 Transcription Factor Staining Buffer Set (00-5523-00, Thermo Fisher Scientific). Cells were recorded on a four-laser Attune Nxt flow cytometer (Thermo Fisher Scientific) or sorted on a three-laser FACSAria II (BD Biosciences). Data were analyzed using FlowJo software (Treestar).

10.1136/jitc-2021-003293.supp1 Supplementary data

### Adoptive T cell transfer

OT-I CD8^+^ T cells were purified from spleens by negative selection magnetic-activated cell sorting (130-104-075, Miltenyi Biotec). For proliferation experiments, OT-I CD8^+^ T cells were labeled with 5 µM CTV (C34557, Thermo Fisher Scientific). 0.5×10^6^ OT-I CD8^+^ T cells in 200 µL PBS were intravenously (i.v.) transferred.

### Anti-SIINFEKL cytotoxicity and anti-interferon-γ ELISpot assay

Splenocytes were suspended in RPMI-1640 + 10% FBS. Half of the splenocytes were pulsed with SIINFEKL (ovalbumin, OVA_257–264_) (10 µg/mL) (AS-60 193-1, AnaSpec) for 2 hour at 37°C, 5% CO_2_ and remaining cells were left unloaded. Cells loaded with SIINFEKL were labeled with 5 µM CTV and unloaded cells were labeled with 500 nM CTV. Splenocytes were pooled 1:1 and transferred i.v. (10^7^ cells/mouse in 200 µL PBS). Tumor-draining lymph nodes (TDLNs) from recipient mice were processed to single cells and seeded (2.5×10^5^ cells/well) for 72 hours in RPMI-1640 + 10% FBS+SIINFEKL peptide (5 µg/mL) in a 96-well plate, precoated overnight (4°C) with anti-interferon-γ (IFN-γ) antibody (CT317-T2, U-CyTech Biosciences). ELISpots were developed according to the manufacturer’s instructions and the remainder of cells was recorded via flow cytometry.

### Antibodies in depletion and inhibition *in vivo* experiments

For depletion of CD8^+^ T cells, 250 µg of anti-mouse CD8α monoclonal antibody (clone YTS 169.4, BP0117, Bio-Connect) was intraperitoneally (i.p.) administered. For inhibition of IFN-γ activity, 500 µg of a neutralizing anti-mouse IFN-γRI monoclonal antibody (clone GR-20, BE0029, Bio-Connect) was i.p. administered.

### CD8^+^ T cell sorting and RNA isolation

CD8^+^ T cells were sorted (4°C) from mixed cell populations and captured in RLT Plus lysis buffer + β-mercaptoethanol. RNA was isolated following the manufacturer’s instructions (RNeasy Plus Micro Kit, 74034, Qiagen). Concentration, quality and RNA integrity was assessed using an Agilent 2100 Bioanalyzer (Agilent). Samples with RIN ≥ 8 and 280/260 and 260/230 values > 1.8 were used for library preparation.

### T cell receptor sequencing, data processing and analysis

The sequencing library was constructed using the Qiagen QIAseq Mouse T cell receptor (TCR) Panel Immune Repertoire RNA Library Kit (Qiagen) according to the manufacturer’s instructions. More detail can be found in online supplemental methods.

### *Ex vivo* restimulation for detection of intracellular cytokine production

Splenocytes were suspended in RPMI-1640 + 10% FBS + SIINFEKL peptide (10 µg/mL) and incubated for 2 hours at 37°C, 5% CO_2_. After 2 hours, brefeldin A solution (1000 x) (00-4506-51, Thermo Fisher Scientific) was added and samples were further incubated at 37°C, 5% CO_2_ overnight.

### Multiplex cytokine analysis

Blood was sampled from the tail vein, collected in EDTA-coated microcuvette tubes (Sarstedt) and centrifuged at 14 000xg for 10 min at 4°C. Cytokine and chemokine concentrations were determined with the Cytokine and Chemokine Convenience 36-Plex Mouse ProcartaPlex Panel 1A (EPXR360-26092-901, Thermo Fisher Scientific) following the manufacturer’s instructions.

### Statistical analyses and data presentation

Statistical analyses were performed using the GraphPad Prism V.9 software (GraphPad Software). Data are presented as mean ± SEM in all experiments, unless stated otherwise. The number of independent biological replicates or the number of individual mice has been indicated as ‘*n’*. Statistical significance was throughout defined as p < 0.05. More detail can be found in online supplemental methods.

## Results

### A therapeutic cancer vaccine supplemented with CD8α ALN-1 slows down tumor growth without evoking systemic toxicity

**Figure 1:**
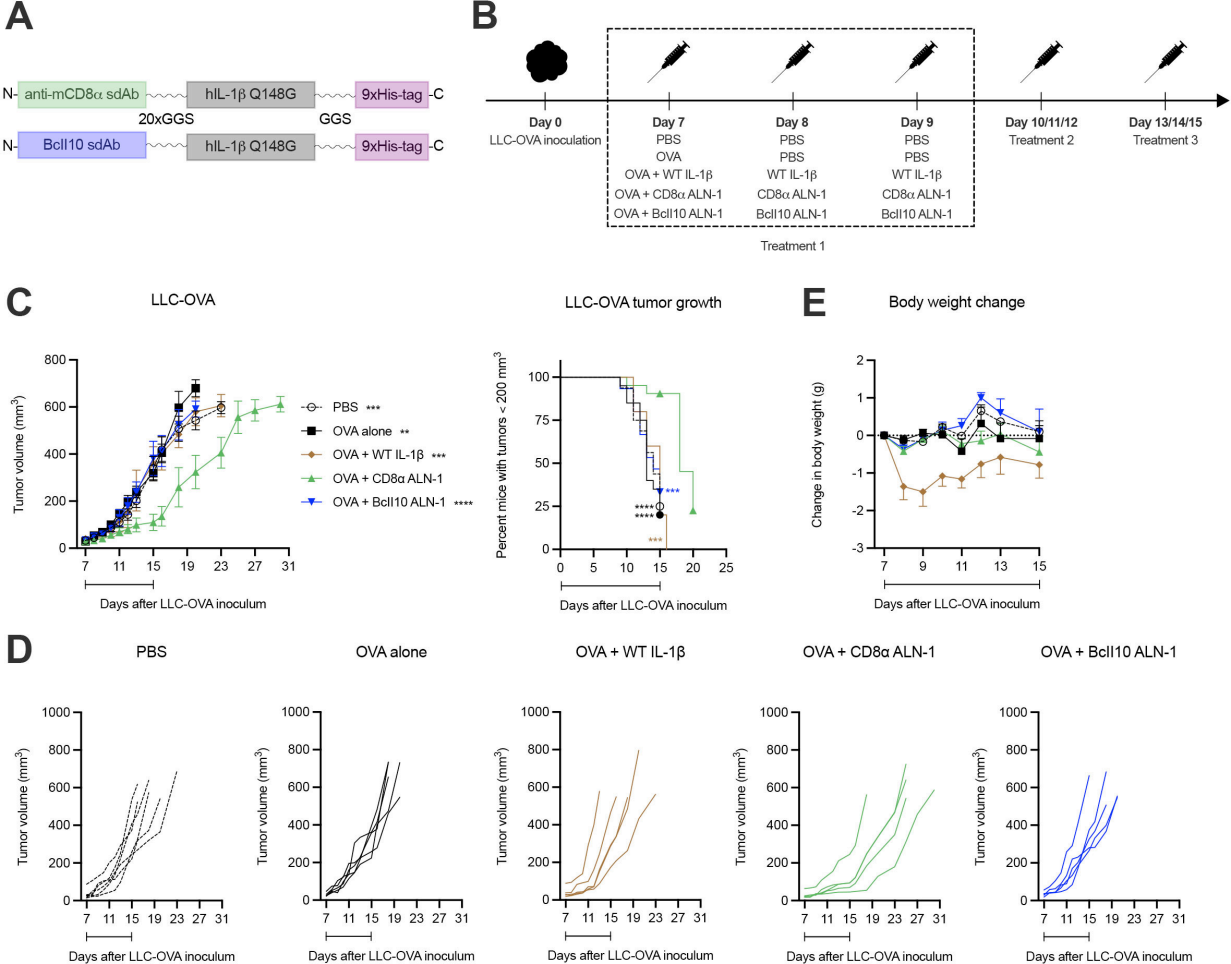
A therapeutic cancer vaccine supplemented with CD8α ALN-1 slows down tumor growth without evoking systemic toxicity. (A) Molecular design of IL-1β AcTakine and BcII10 control. (B) Mice were inoculated with LLC-OVA and treated with OVA (100 µg) or not (PBS). This was combined with treatments with PBS, WT IL-1β (5 µg), CD8α ALN-1 (10 µg) or untargeted BcII10 ALN-1 (10 µg). (C) LLC-OVA tumor growth over time for different treatment conditions (left). Data points represent the mean ± SEM of a representative out of four independent experiments with *n*=6 (PBS) or *n*=5 mice/group. Kaplan-Meier curves demonstrating the required time to reach tumor volumes exceeding 200 mm^3^ (right). Data points represent the mean of pooled data from four independent experiments. (D) LLC-OVA tumor growth for individual treatment conditions. Shown is a representative out of four independent experiments. (E) Change in body weight for different treatment conditions. Data points represent the mean ± SEM of a representative out of four independent experiments with *n*=6 (PBS) or *n*=5 mice/group. Intervals below the curves indicate the treatment period. **p<0.01, ***p<0.001, ****p<0.0001; by two-way ANOVA with Sidak’s multiple comparisons test (C) (left) or by log-rank (Mantel-Cox) testing (C) (right). ANOVA, analysis of variance; IL-1β, interleukin-1β; LLC, Lewis lung carcinoma; OVA, ovalbumin; WT, wild-type.

We first assessed the efficacy and safety of a cancer vaccine adjuvanted with CD8α ALN-1. In order to obtain this AcTakine, we first introduced the Q148G mutation in human IL-1β, which renders a cytokine with low biological activity that can be completely restored on its delivery by a high-affinity sdAb.^26^ Mice with s.c. inoculated LLC that stably expresses the OVA model antigen (LLC-OVA) were treated i.p. with PBS or OVA, either alone or combined with WT IL-1β, CD8α ALN-1 or untargeted BcII10 ALN-1 (figure 1A, B). Treatment with OVA + CD8α ALN-1 caused a significant delay in tumor growth over the treatment period (figure 1C, D), however, tumor control was transient and volumes rapidly caught up with lesion sizes observed in controls on treatment discontinuation. Importantly, this effect depended on CD8α targeting, as neither treatment with WT IL-1β or BcII10 ALN-1 had antitumor effect. Repeated administration of CD8α ALN-1 was free of measurable toxicity, whereas equivalent amounts of WT IL-1β caused body weight loss and gravely impacted the well-being of mice (figure 1E). Systemic delivery of CD8α ALN-1 can thus safely delay tumor growth over the treatment period by targeting CD8α^+^ cells.

### The antitumor effect of CD8α ALN-1 depends on activation of the endogenous CD8^+^ T cell response

**Figure 2:**
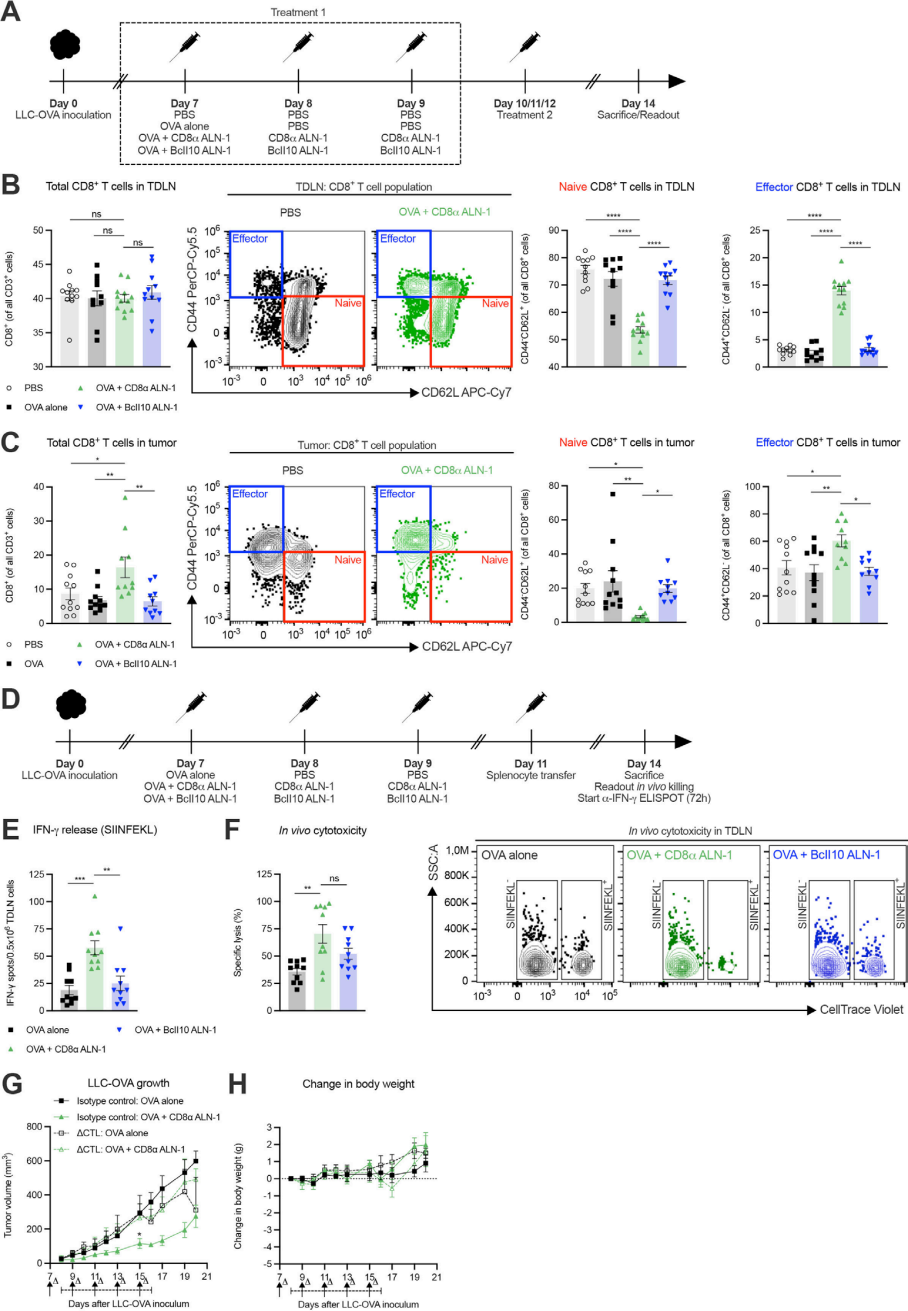
The antitumor effect of CD8α ALN-1 depends on activation of endogenous CD8^+^ T cell immune responses. (A) Mice inoculated with LLC-OVA received OVA (100 µg) or not (PBS) and were treated with PBS, CD8α ALN-1 (10 µg) or BcII10 ALN-1 (10 µg). (B, C) Frequencies of total, naive and effector CD8^+^ T cells in TDLN (B) and tumor tissue (C). Bars represent the mean ± SEM of a pool of two independent experiments with *n*=12 mice/group combined with representative flow cytometry dot plots. (D) Mice inoculated with LLC-OVA received OVA alone (100 µg), OVA + CD8α ALN-1 (10 µg) or OVA + BcII10 ALN-1 (10 µg) and splenocyte transfer. (E) IFN-γ release after splenocyte *ex vivo* restimulation, measured by ELISpot. (F) Cytotoxicity toward SIINFEKL^+^ cells with representative flow cytometry dot plots (right). Bars represent the mean ± SEM of a pool of two independent experiments with *n*=10 mice/group combined. (G) Growth of LLC-OVA in mice treated with a CTL-depleting monoclonal antibody (250 µg) or an isotype control (250 µg). (H) Change in body weight of the mice shown in (G). Antibody administration is indicated by arrows, the treatment period is designated by an interval. Data points represent the mean ± SEM of an experiment with *n*=5 mice/group. *p<0.05, **p<0.01, ***p<0.001, ****p<0.0001; ns, *p*≥0.05 by one-way ANOVA with Tukey’s multiple comparisons test in (B, C, E, F) or by two-way ANOVA with Sidak’s multiple comparisons test in (G). See also online supplemental figures 1 and 2). ANOVA, analysis of variance; IFN-γ, interferon-γ; LLC, Lewis lung carcinoma; ns, not significant; OVA, ovalbumin; TDLN, tumor-draining lymph node.

Next, we studied the CD8^+^ T cell response in the TDLN and LLC-OVA tumor tissue after treatment (figure 2A). While we did not find statistically significant differences in the relative amount of CD8^+^ T cells in the TDLN, mice treated with OVA + CD8α ALN-1 showed reduced frequencies of naive CD8^+^ T cells compared with controls (figure 2B, see online supplemental figure 1A for the gating strategy). Accordingly, this corresponded with an increase in the relative amount of effector CD8^+^ T cells. In tumors, treatment with OVA + CD8α ALN-1 caused an increase in the fraction of CD8^+^ T cells, a decrease of naive CD8^+^ T cells and higher frequencies of effector CD8^+^ T cells compared with controls (figure 2C, see online supplemental figure 1B for the gating strategy).

We then measured the capacity of CD8^+^ T cells to release IFN-γ after *ex vivo* restimulation with TAA and kill target cells in vivo (figure 2D). Upon *ex vivo* restimulation of TDLN cells with SIINFEKL (OVA_257-264_), we recorded more IFN-γ release after treatment with OVA + CD8α ALN-1 (figure 2E). Concomitantly, we observed enhanced specific cytolytic activity in mice treated with OVA + CD8α ALN-1 (figure 2F, see online supplemental figure 2 for the gating strategy). Collectively, treatment with CD8α ALN-1 stimulates host CD8^+^ T cells in the TDLN and tumor to acquire a favorable effector phenotype and execute effector functionality in response to TAA encounter.

We analyzed the importance of CD8^+^ T cells in the CD8α ALN-1-mediated antitumor effect by depletion of cytotoxic T lymphocytes (CTLs) prior to and during treatment. The partial control over tumor growth on treatment with OVA + CD8α ALN-1 was completely lost on CTL depletion (figure 2G). Again, repeated delivery of CD8α ALN-1 was not associated with measurable toxicity (figure 2H). These data demonstrate the necessity of CD8^+^ T cells during the antitumor response initiated by CD8α ALN-1.

### TCR repertoire analysis reveals increased host CTL diversity induced by CD8α ALN-1

**Figure 3:**
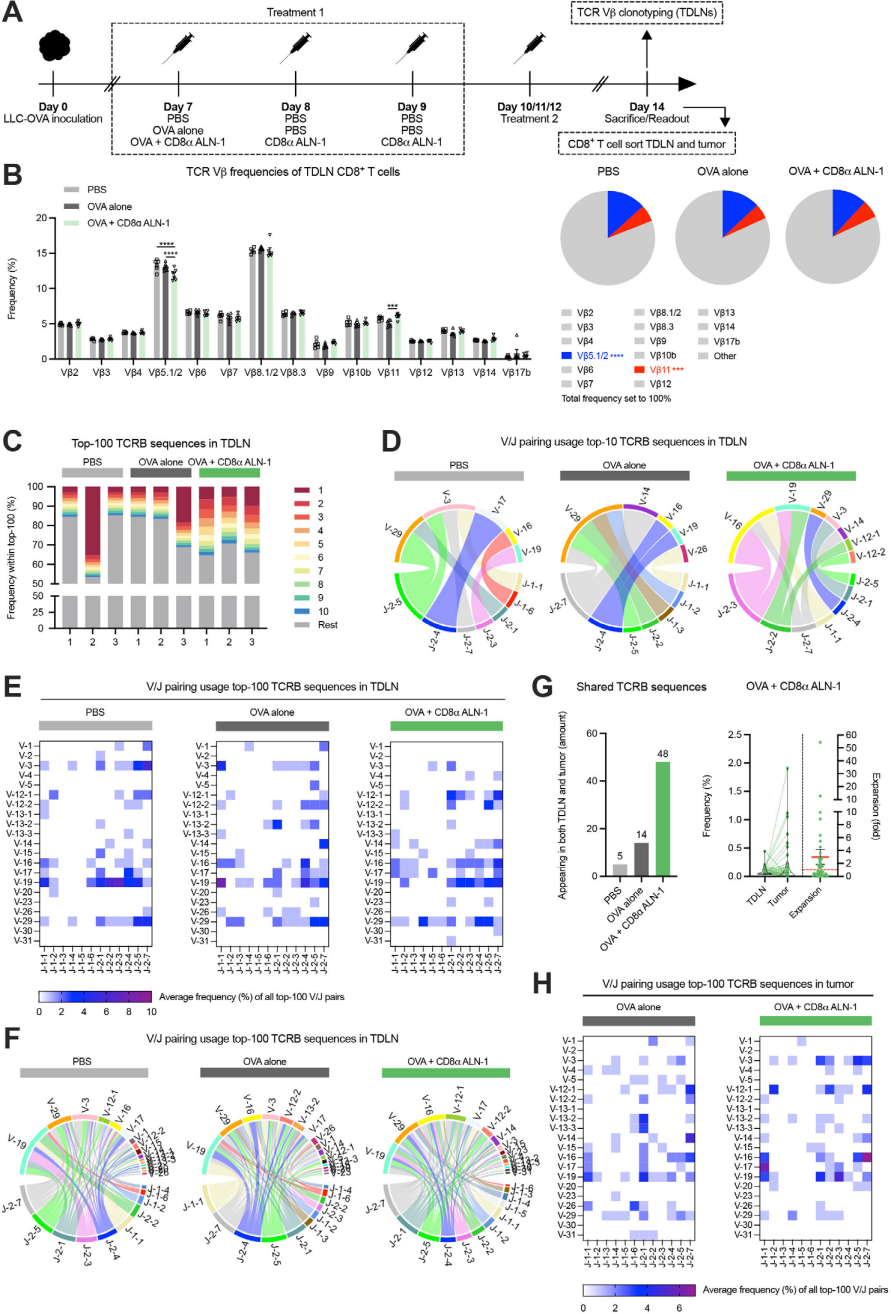
TCR repertoire analysis reveals increased host CTL diversity induced by CD8α ALN-1. (A) Mice inoculated with LLC-OVA received treatment with OVA (100 µg) or not (PBS) in combination with PBS or CD8α ALN-1 (10 µg). (B) Frequencies of 15 different TCR Vβ clonotypes within the CD8^+^ T cell population (left) with parts-of-whole (right) representing cumulative frequencies of analyzed clonotypes. Remaining unannotated clonotypes are indicated as ‘other’, colored fractions indicate clonotypes for which statistically significant changes were observed. Bars represent the mean ± SEM of an experiment with *n*=6 mice/group. ***p<0.001, ****p<0.0001 by two-way ANOVA with Sidak’s multiple comparisons test. (C) Frequency of the top-10 most abundant TCRB sequences (stacked bars in color) within the top-100 most abundant TDLN TCRB sequences. The same color does not necessarily represent the same clonotype in the different bars. (D–F) V/J pairing usage within the top-10 (D) and the top-100 (E) and (F) most abundant TDLN TCRB sequences. (G) Amount of sequences from the top-100 TDLN TCRB sequences that are retrieved in matched tumors (left). Relative frequencies of shared sequences in the TDLN and tumor from mice treated with OVA + CD8α ALN-1. Relative expansion of shared sequences in the tumor with the mean relative expansion (red full line) ± SEM; red dotted line indicates relative expansion of 1 (right). (H) V/J pairing usage within the top-100 most abundant TCRB sequences in the tumor. See also online supplemental figures 3–5 and table 2. ANOVA, analysis of variance; CTL, cytotoxic T lymphocytes; LLC, Lewis lung carcinoma; OVA, ovalbumin; TCR, T cell receptor; TDLN, tumor-draining lymph node.

We assessed whether treatment with CD8α ALN-1 mediates clonal expansion of host T cells. We first used a method that detects changes in murine TCR repertoires via flow cytometry-based TCR Vβ analysis (figure 3A, see online supplemental figure 3 for the gating strategy).^27^ Treatment with OVA + CD8α ALN-1 caused significant, although modest, changes in frequencies of different CD8^+^ T cell TCR Vβ clonotypes (Vβ5.1/2 and Vβ11) compared with OVA alone (figure 3B). Of note, decrease of the dominant TCR Vβ5.1/2 clonotype was compensated by small increases in various other clonotypes (including TCR Vβ9, Vβ11, Vβ13 and Vβ14; summarized in online supplemental table 2) that did not reach statistical significance.

Next, we performed TCR sequencing to investigate effects on the T cell repertoire in more depth. We sorted CD8^+^ T cells from the TDLN and LLC-OVA tumor of mice treated with PBS, OVA alone or OVA + CD8α ALN-1 and isolated RNA for bulk TCR sequencing (figure 3A, see online supplemental figure 4A and B for the gating strategies). Host TCRB sequences in TDLN revealed clear expansion of numerous CD8^+^ T cell clones after treatment with OVA + CD8α ALN-1 (figure 3C and online supplemental figure 5A). This robust expansion occurred in all mice and indicates epitope spreading, further evidenced by the top-10 clones covering ≥30% of the top-100. This effect was absent in control mice, where either no clonal expansion or strong dominance of one established clone could be observed. After treatment with CD8α ALN-1, the top-10 most abundant TCRB sequences in TDLN also appeared more diverse in terms of V/J pairing usage (figure 3D). Regarding the top-100 TDLN clones, average V/J pairing usage frequencies revealed spread of the CTL response over numerous smaller TCRB V/J pairs in mice treated with CD8α ALN-1, whereas T cells isolated from controls were enriched in a smaller selection of V/J combinations (figure 3E, F and online supplemental figure 5B). Examination of matched tissues learned that more clones from the TDLN top-100 could be retrieved in tumors upon treatment with OVA + CD8α ALN-1 and several of these clones also expanded (figure 3G). Additionally, average V/J pairing usage frequencies in the tumor show oligoclonal expansion of CD8^+^ T cells for certain TCRB V/J sequences after treatment with OVA + CD8α ALN-1 (figure 3H and online supplemental figure 5C–F). Collectively, these data present evidence that the antitumor response mediated by CD8α ALN-1 correlates with clonal expansion (online supplemental figure 5G and H) and increased TCR repertoire diversity in the TDLN and the tumor tissue, implying epitope spreading.

### Treatment with CD8α ALN-1 empowers activation and proliferation of adoptively transferred tumor-specific CD8^+^ T cells

**Figure 4:**
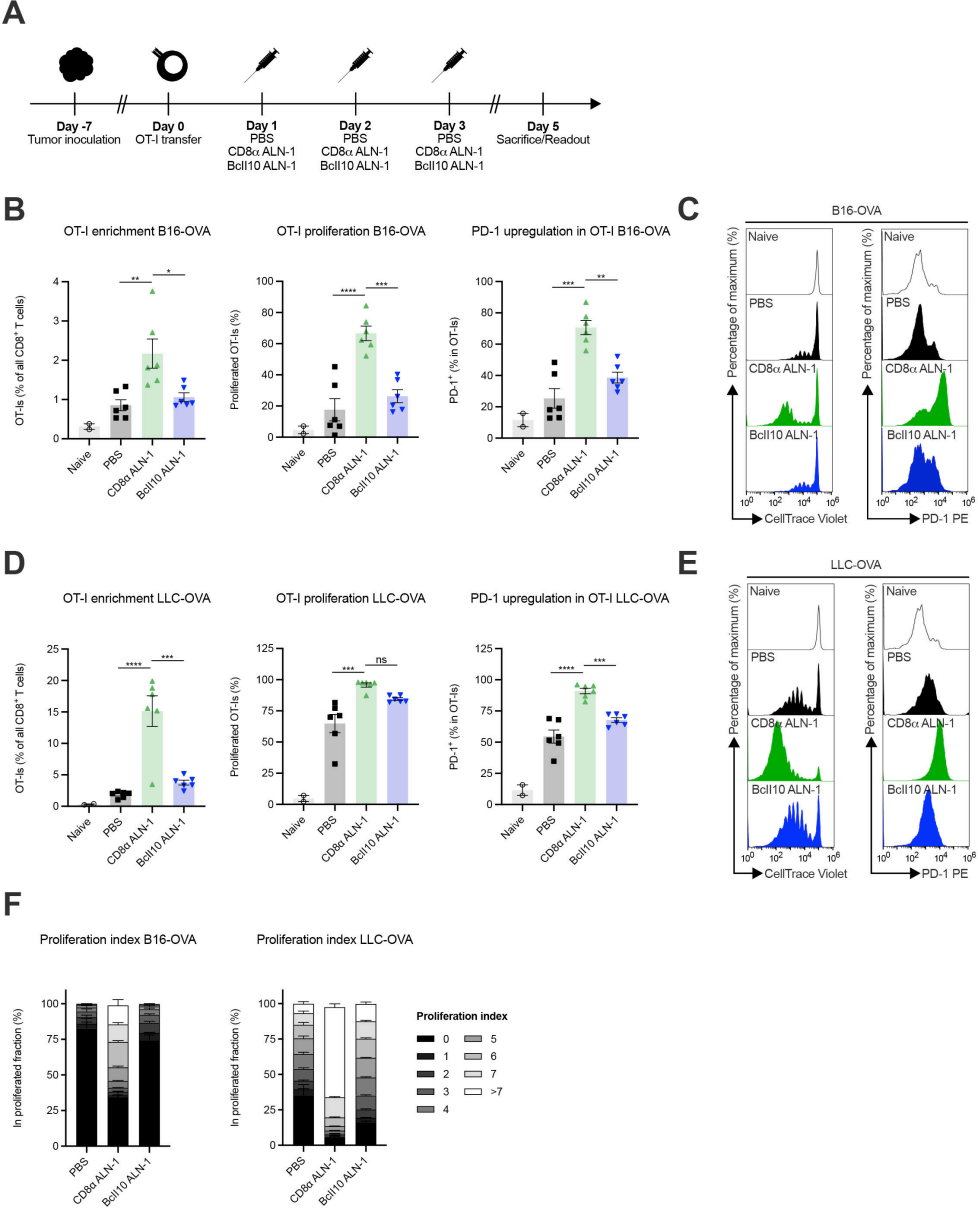
Treatment with CD8α ALN-1 empowers activation and proliferation of adoptively transferred tumor-specific CD8^+^ T cells. (A) Mice inoculated with B16- or LLC-OVA received OT-I CD8^+^ T cells and treatments with PBS, CD8α ALN-1 (10 μg) or BcII10 ALN-1 (10 µg). (B–E) Frequencies of OT-I CD8^+^ T cells within the total CD8^+^ T cell population in TDLN (left), percentage of total proliferated OT-I CD8^+^ T cells (middle) and expression of PD-1 on OT-I CD8^+^ T cells (right) in B16- (B) and LLC-OVA (D) tumor-bearing mice. Bars represent the mean ± SEM of a representative of two independent experiments with *n*=2 (naive) or *n*=6 mice/group. Representative flow cytometry histograms displaying OT-I CD8^+^ T cell proliferation (left) and PD-1 upregulation (right) in TDLN of B16- (C) and LLC-OVA (E) tumor-bearing mice. (F) Stacked histograms summarize the proliferation index of OT-I CD8^+^ T cells in B16- (left) and LLC-OVA (right) tumor-bearing mice. Individual stacks represent the mean percentages of proliferating OT-I CD8^+^ T cells in certain stages of cell division ± SEM. Shown is a representative of two independent experiments with *n*=6 mice/group. *p<0.05, **p<0.01, ***p<0.001, ****p<0.0001; ns, p≥0.05 by one-way ANOVA with Tukey’s multiple comparisons test. See also online supplemental figures 6 and 7. ANOVA, analysis of variance; LLC, Lewis lung carcinoma; ns, not significant; OVA, ovalbumin; TDLN, tumor-draining lymph node.

Next, we examined whether CD8α ALN-1 empowers activation and proliferation of adoptively transferred OVA-specific CD8^+^ T cells in response to TAA encounter (figure 4A). In mice with established B16- or LLC-OVA tumors, CD8α ALN-1 strongly promoted OT-I CD8^+^ T cell accumulation, proliferation and activation, measured as enhanced PD-1 surface expression, in the TDLN compared with control mice (figure 4B–E, see online supplemental figure 6 for the gating strategy). Of note, more pronounced effects were consistently observed in LLC-OVA^+^ mice. The proliferation index, defined as the fraction of OT-I CD8^+^ T cells in a certain stage of cell division, shows that treatment with CD8α ALN-1 not only engaged more OT-I CD8^+^ T cells to initiate proliferation, but these cells also completed more divisions (figure 4F). This is especially apparent in LLC-OVA^+^ mice, where treatment with CD8α ALN-1 pushed proliferation to the latest stages. We could measure comparable effects in the pmel-model, where CD8^+^ T cells with reactivity toward the gp100 antigen are transferred in mice that carry B16 gp100^+^ melanomas (online supplemental figure 7). Together, these data show that CD8α ALN-1 acts as a potent cellular adjuvant that enhances accumulation, proliferation and activation of adoptively transferred tumor-specific CD8^+^ T cells in response to TAA encounter.

### CD8α ALN-1 enhances the efficacy of ATCT by endorsing effector CD8^+^ T cell generation in both TDLN and TME

**Figure 5:**
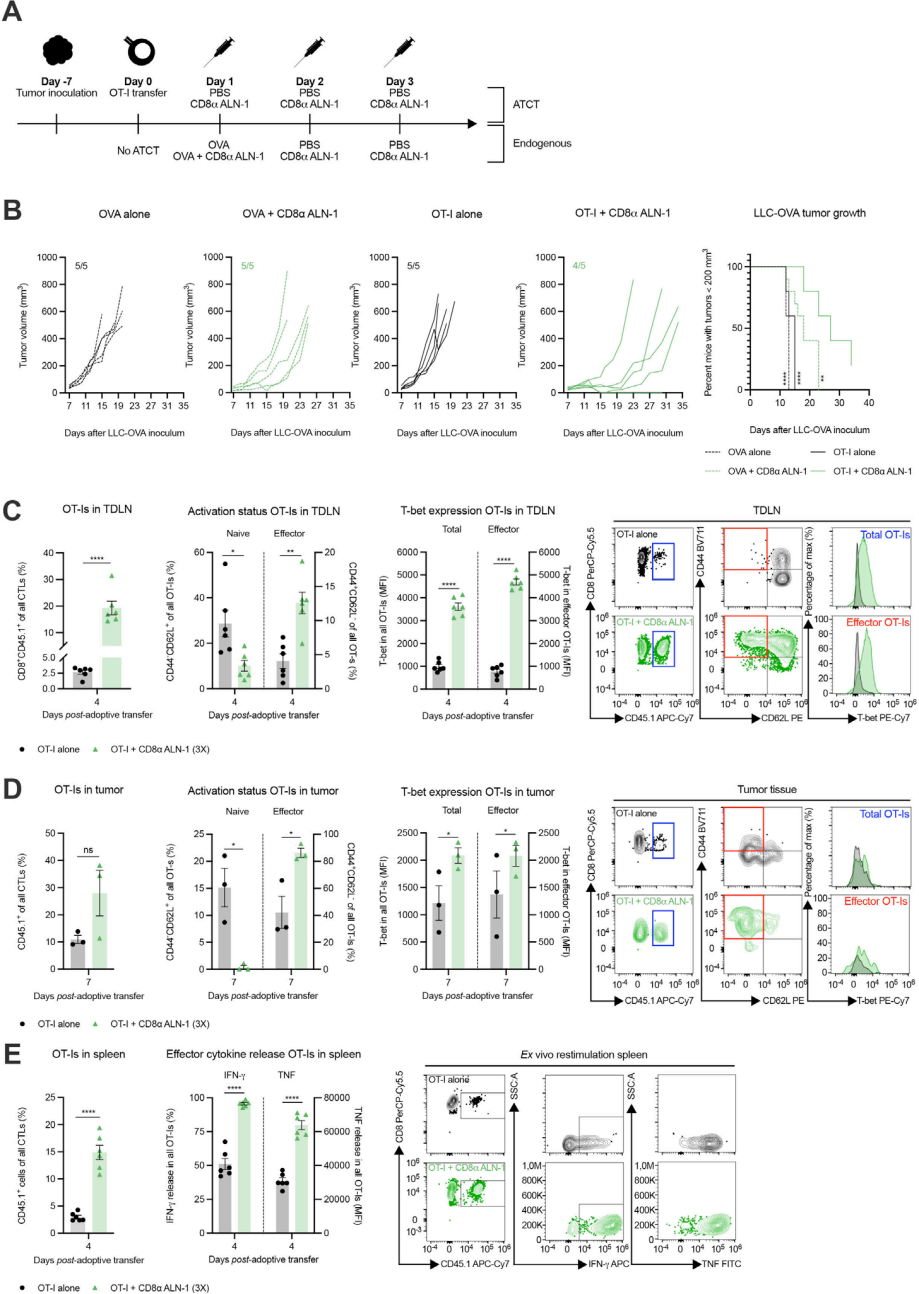
CD8α ALN-1 enhances the efficacy of adoptive T cell transfer by endorsing effector CD8^+^ T cell generation in both tumor-draining lymph node and tumor microenvironment. (A) Mice carrying established LLC-OVA tumors received OT-I CD8^+^ T cells and treatment with PBS or CD8α ALN-1 (10 µg). Controls did not receive OT-I CD8^+^ T cells and were treated with OVA alone (100 µg) or combined with CD8α ALN-1 (10 µg). (B) LLC-OVA tumor growth. Lines represent individual mice. Shown is a representative of two independent experiments with *n*=5 mice/group. Kaplan-Meier curves demonstrate time to reach tumor volumes exceeding 200 mm^3^. Pooled data from two independent experiments with *n*=10 mice/group combined. (C, D) Frequencies of OT-I CD8^+^ T cells within total CD8^+^ T cells, naive and effector phenotypes within OT-I CD8^+^ T cells and T-bet expression within the total and effector OT-I CD8^+^ T cell population in the TDLN (C) and tumor tissue (D) with representative flow cytometry dot plots. (E) Frequencies of OT-I CD8^+^ T cells within the total CD8^+^ T cell population and production of IFN-γ and TNF by OT-I CD8^+^ T cells following *ex vivo* splenocyte restimulation with representative flow cytometry dot plots. Bars represent the mean ± SEM of an experiment with *n*=6 mice/group (for TDLN and spleen samples) or *n*=3 mice/group (for tumor samples, as two mice were pooled together). *p<0.05, **p<0.01, ****p<0.0001; ns, p≥0.05 by log-rank (Mantel-Cox) testing (B) or by unpaired Student’s t-test (C–E). See also online supplemental figure 8 and 9. IFN-γ, interferon-γ; LLC, Lewis lung carcinoma; ns, not significant; OVA, ovalbumin; TDLN, tumor-draining lymph node; TNF, tumor necrosis factor.

We next examined whether combination of CD8α ALN-1 with OT-I CD8^+^ T cell transfer could improve the therapeutic window of ATCT. Control mice did not receive ATCT and were treated with OVA, either alone or combined with CD8α ALN-1 (figure 5A). Only three CD8α ALN-1 administrations strongly empowered the therapeutic efficiency of ATCT (figure 5B). Compared with mice that received ATCT alone, the combination with CD8α ALN-1 significantly increased the relative amount of OT-I CD8^+^ T cells and the fraction of effector OT-I CD8^+^ T cells in the TDLN (figure 5C, see online supplemental figure 8A for the gating strategy). As expected, this corresponded with an increase in T-bet expression in both the total and the effector OT-I CD8^+^ T cell population in the TDLN of CD8α ALN-1-treated mice compared with controls.

Also in tumors, combination of ATCT with CD8α ALN-1 yielded larger amounts of effector OT-I CD8^+^ T cells and enhanced T-bet expression in both the total and the effector OT-I CD8^+^ T cell population (figure 5D). After *ex vivo* splenocyte restimulation, we observed more release of IFN-γ and TNF by OT-I CD8^+^ T cells from mice that received ATCT in combination with CD8α ALN-1 (figure 5E). Comparable effects were observed when the total population of CD8^+^ T cells in the TDLN, tumor tissue and spleen of LLC-OVA^+^ mice was considered (online supplemental figure 9A–C, see online supplemental figure 8B) for the gating strategy). In the tumor, treatment with CD8α ALN-1 reduced the relative amount of CD8^+^ T cells with a dysfunctional profile, characterized by expression of CD101 and CD38, and enhanced expression of the transcription factor TCF-1 (online supplemental figure 9D).^28^ These latter results are of particular interest in light of recent findings that have linked stem cell-like properties to TCF-1^+^ CD8^+^ T cells and correlated those with a positive clinical response to checkpoint inhibition.^29 30^ In conclusion, CD8α ALN-1 empowers ATCT efficiency by enhancing accumulation of functionally potent effector CD8^+^ T cells in the TDLN and the tumor.

### cDC1s are dispensable for the cellular adjuvant effect of CD8α ALN-1, but lower the threshold for efficient CD8^+^ T cell proliferation

**Figure 6:**
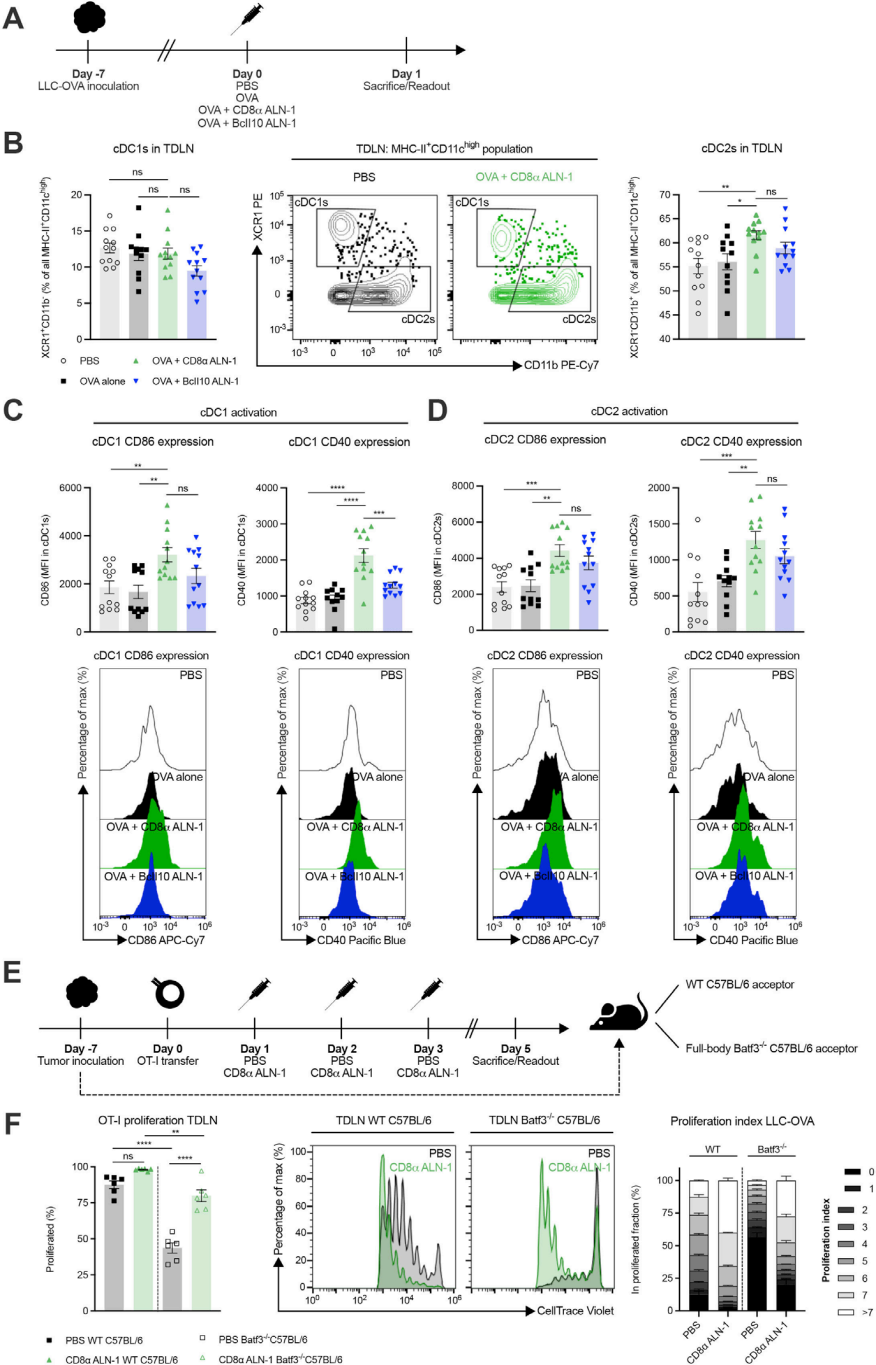
cDC1s are dispensable for the cellular adjuvant effect of CD8α ALN-1, but lower the threshold for efficient CD8^+^ T cell proliferation. (A) Mice with established LLC-OVA tumors were treated with either PBS, OVA alone (100 µg) or combined with CD8α ALN-1 (10 µg) or untargeted BcII10 ALN-1 (10 µg). (B) Relative amounts of cDC1s (left) and cDC2s (right) within the total cDC population with representative flow cytometry dot plots (middle). (C, D) Expression of the activation markers CD40 (left) and CD86 (right) on cDC1s (C) and cDC2s (D) with representative flow cytometry histograms (bottom). (E) OT-I CD8^+^ T cells were adoptively transferred in WT or full-body Batf3^-/-^ C57BL/6 mice carrying established LLC-OVA tumors. Acceptor mice received treatments with either PBS or CD8α ALN-1 (10 µg). (F) Fraction of proliferated OT-I CD8^+^ T cells (left) with representative flow cytometry histograms (middle). Stacked histograms summarize the proliferation index of OT-I CD8^+^ T cells in WT or full-body Batf3^-/-^ tumor-bearing acceptor mice (right). Bars represent the mean ± SEM of a pool of two independent experiments with *n*=12 mice/group combined (B–D) or an experiment with *n*=6 mice/group (F). *p<0.05, **p<0.01, ***p<0.001, ****p<0.0001; ns, *p*≥0.05 by one-way ANOVA with Tukey’s multiple comparisons test. See also online supplemental figures 10–13. ANOVA, analysis of variance; LLC, Lewis lung carcinoma; ns, not significant; OVA, ovalbumin; TDLN, tumor-draining lymph node; WT, wild-type.

As murine cDC1s are CD8α^+^ and can thus be a target for CD8α ALN-1, we looked into their activation status in the TDLN (figure 6A). While the relative amount of cDC1s within the total cDC population remained unaltered, we measured a significant increase in cDC2 percentages following treatment with OVA + CD8α ALN-1 compared with PBS and OVA alone (figure 6B, see online supplemental figure 10 for the gating strategy). cDC1s and cDC2s both upregulated the activation markers CD40 and CD86 following treatment with OVA + CD8α ALN-1 (figure 6C, D). Of note, no significant difference in CD86 expression on cDC1s and CD40/CD86 expression on cDC2s was found between treatment with OVA + CD8α ALN-1 and OVA + untargeted BcII10 ALN-1.

Because cDC1s are critical for activation of CD8^+^ T cells, we evaluated their necessity for the adjuvant effect of CD8α ALN-1 (see online supplemental figure 11 for the gating strategy). Both in WT and full-body Batf3^-/-^ mice, treatment with OVA + CD8α ALN-1 significantly increased the frequency of effector CD8^+^ T cells in the TDLN and their expression of T-bet and Eomes (online supplemental figure 12A nd B). Absence of cDC1s reduced T-bet expression in CD8^+^ T cells (online supplemental figure 12B) and increased the fraction of Eomes^+^T-bet^dim^ effector CD8^+^ T cells induced upon CD8α ALN-1 delivery (online supplemental figure 12C). Additionally, we transferred OT-I CD8^+^ T cells in WT or full-body Batf3^-/-^ mice and measured proliferation in the TDLN (figure 6E, see online supplemental figure 13A for the gating strategy). In Batf3^-/-^ mice, fewer OT-I CD8^+^ T cells were present in the TDLN (online supplemental figure 13B), yet treatment with CD8α ALN-1 still promoted their proliferation (figure 6F). While treatment with OVA + CD8α ALN-1 does activate cDCs in the TDLN, presence of cDC1s does not seem to be fundamental for its adjuvant effect. However, signals rendered by cDC1s facilitate the CD8^+^ T cell response following treatment with CD8α ALN-1, for instance by lowering the threshold for expansion.

### Strong synergy between CD8α ALN-1 and neovasculature-targeted TNF mediates complete tumor eradication and protective memory against secondary tumor challenge

**Figure 7:**
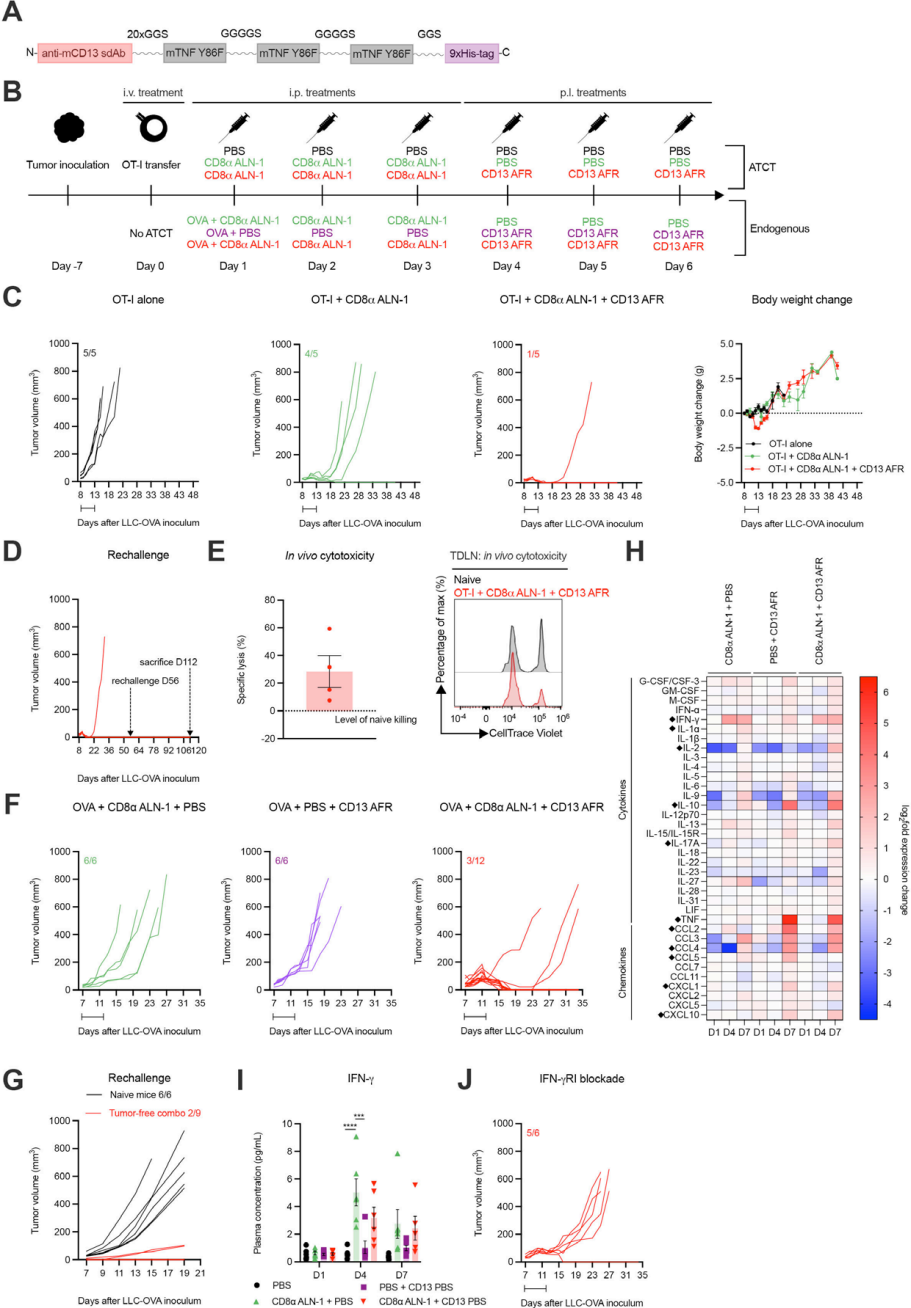
Strong synergy between CD8α ALN-1 and neovasculature-targeted TNF mediates complete tumor eradication in the absence of systemic toxicity and allows for immunological protection against secondary tumor challenge. (A) Molecular TNF AcTakine design. (B) Mice with LLC-OVA tumors received OT-I CD8^+^ T cell transfer (top) or not (bottom) and were treated with PBS or CD8α ALN-1 (10 µg) in combination with PBS or CD13 AFR (50 µg) (top). Mice not treated with ATCT (bottom) received OVA (100 µg) combined with treatments described above (top). (C) LLC-OVA tumor growth (left) and change in body weight (right) (ATCT). Lines represent individual mice. Data points represent the mean ± SEM of an experiment with *n*=5 mice/group. (D) LLC-OVA tumor growth (ATCT, rechallenge). (E) Cytotoxicity towards SIINFEKL^+^ cells. Bar represents the mean ± SEM of an experiment with *n*=4 mice/group (left) with representative flow cytometry histograms (right). (F) LLC-OVA tumor growth (without ATCT). Lines represent individual mice of an experiment with *n*=6 or 12 mice/group. (G) LLC-OVA tumor growth (without ATCT, rechallenge). (H) Heat map of log_2_-fold changes in plasma cytokine and chemokine levels. Group means of an experiment with *n*=6 mice/group. Statistically significant differences are indicated with a diamont icon. (I) Plasma IFN-γ concentration. Bars represent the mean ± SEM of an experiment with *n*=6 mice/group. ***p<0.001, ****p<0.0001; ns, p≥0.05 by one-way ANOVA with Tukey’s multiple comparisons test. (J) LLC-OVA tumor growth. Mice were treated with an anti-mouse IFN-γRI antibody (500 µg) every 48 hours starting 1 day before the first CD8α ALN-1 delivery. Intervals indicate the treatment periods. See also online supplemental figures 14–16. AcTakine, Activity-on-Target cytokine; ANOVA, analysis of variance; ATCT, adoptive T cell transfer; IFN-γ, interferon-γ; IL, interleukin; LLC, Lewis lung carcinoma; ns, not significant; OVA, ovalbumin; TNF, tumor necrosis factor.

Recently, we developed an AcTakine that targets TNF activity (AcTafactor/AFR) to tumor neovasculature via an anti-mouse CD13 sdAb (figure 7A).^31^ In this way, the ability of TNF to activate tumor endothelial cells and consequentially promote CTL influx into the tumor can be exploited in the absence of toxicities and undesired off-target side effects. Of note, AFR incorporates a murine cytokine mutant, as TNF activity, in sharp contrast with that of IL-1β, is highly species-specific. Treatment with CD13 AFR efficiently eradicated large established tumors in different combinations, including ATCT of human CD70 CAR-T cells, without eliciting the toxicities observed with WT TNF. We were, therefore, driven to examine whether combination with CD13 AFR delivery could improve the efficacy of CD8α ALN-1 treatment.

First, we tried to further improve the efficacy of OT-I CD8^+^ T cell adoptive transfer combined with CD8α ALN-1 treatment by additional delivery of CD13 AFR (figure 7B, upper part). Addition of CD13 AFR allowed for complete eradication of established LLC-OVA tumors with limited loss in body weight (approximately 5%) (figure 7C). Characteristic for this antitumor effect is strong and complete tumor necrosis, allowing mice to remain tumor-free for >50 days after inoculation. Importantly, these mice were fully protected against secondary tumor challenge, which correlated with CD8^+^ T cell-mediated cytotoxicity (figure 7D, E). We next combined treatment with OVA + CD8α ALN-1 with CD13 AFR administration, without prior ATCT (figure 7B, bottom part). In this completely endogenous model, the combination induced full tumor eradication in 9/12 mice (figure 7F). Whereas naive mice could not control tumor growth after rechallenge, 7/9 mice previously treated with CD8α ALN-1 and CD13 AFR were protected (figure 7G). Comparable results could be obtained in the B16 melanoma model (online supplemental figure 14).

In order to mechanistically understand this AcTakine synergy, we sampled blood from LLC-OVA^+^ mice and analyzed cytokine and chemokine plasma levels using a Luminex multiplex assay (online supplemental figure 15A). Treatment with CD8α ALN-1 significantly enhanced IFN-γ levels compared with PBS treatment (figure 7H, I). IFN-γ has been described to enhance sensitivity of tumor endothelial cells for TNF activity and could therefore be an important mediator of this synergy.^31^ Indeed, on systemic administration of an anti-mouse IFN-γRI antibody in LLC-OVA^+^ mice, treatment with CD8α ALN-1 combined with CD13 AFR only induced complete tumor necrosis in one in five mice (figure 7J). Multiplex analysis further revealed that only CD13 AFR increases plasma levels of different chemokines, including CCL2, CCL4, CCL5, CXCL1 and CXCL10, and IL-10 (figure 7H and online supplemental figures 15B and 16). Combination of CD8α ALN-1 and CD13 AFR enhanced IL-2 and IL-17A release compared with PBS treatment or monotherapy. In summary, CD8α ALN-1 promotes proliferation and effector functionality of antitumor CD8^+^ T cells and synergizes with CD13 AFR to mediate complete tumor eradication, allowing protection against secondary challenge. This AcTakine combination is empowered by IFN-γ, which is probably released by CD8α ALN-1-activated CD8^+^ T cells and sensitizes the TME for TNF.

## Discussion

The current mantra in the search for stronger and more durable antitumor T cell responses can be summarized as ‘releasing the breaks, pushing the gas pedal’. The former can be achieved by inhibition of immune checkpoints, which represent molecules that keep the immune response ‘in check’ following its initiation. Considerable clinical success has been booked by interfering with the interaction between PD-1 and its ligands. ‘Pushing the gas’, on the other hand, has not yet achieved the same extent of results. Personalized cancer vaccines based on tumor-derived neoantigens could be an important leap forward, but their efficacy will depend on potent adjuvants that are able to drive T cell immunity.

To widen the therapeutic window of cytokines with valuable therapeutic properties, we devised AcTakines, which represent a novel class of immunocytokines with the potential to circumvent the toxicities (*e.g.*, severe body weight loss, influenza-like symptoms and cytokine storm) and unwanted side effects associated with systemic administration of WT cytokines and conventional immunocytokines. Here, we report the use of an IL-1β-based AcTakine as a cellular adjuvant to promote antitumor T cell responses. We previously demonstrated that this AcTakine acts as a potent and safe adjuvant in influenza vaccination by stimulating CD8^+^ T cell effector function and memory differentiation.^26^ Considering the high need for powerful drivers of antitumor immunity, we explored the adjuvant effect of this AcTakine in cancer immunotherapy.

As a monotherapy, CD8α ALN-1 showed a modest effect on tumor growth, which was absent in mice that received WT IL-1β or untargeted BcII10 ALN-1. This demonstrates the advantage of selective delivery of IL-1 activity to CD8α^+^ cells, but despite this, the observed antitumor response was incomplete. Indeed, tumor sizes in mice that received CD8α ALN-1 quickly caught up with those in control animals. Nonetheless, treatment with CD8α ALN-1 massively increased CD8^+^ T cell expansion and shifted the naive/effector-ratio towards the latter in the TDLN and tumor. Moreover, these T cells readily released IFN-γ on *ex vivo* restimulation and exerted potent cytolytic activity *in vivo*, which further endorses their effector phenotype. The importance of CD8^+^ T cells in this antitumor response was confirmed by the loss of the treatment effect following their depletion. We found that delivery of IL-1 activity to CD8α^+^ cells outperformed treatment with WT IL-1β, likely due to circumvention of the cytokine’s protumorigenic properties.^20^ Besides improved antitumor action, we noticed a drastic reduction in toxicity on administration of CD8α ALN-1, which is in accordance with data from our earlier work.^26^

How cancer vaccines will deal with tumor heterogeneity remains an important concern. As tumors are highly polyclonal, the breadth of the CD8^+^ T cell response will be an important determinant of clinical efficacy.^12^ Indeed, clinical studies have demonstrated that patients with the best objective responses to DC vaccination showed evidence of epitope spreading.^32 33^ Earlier work demonstrated that inflammatory cytokines can fine-tune the sensitivity of TCRs, theoretically allowing for a more diverse T cell response in terms of epitope recognition.^34^ Whether this applies for IL-1β activity is currently unknown and therefore, we evaluated the TCR repertoire of CD8^+^ T cells following treatment with CD8α ALN-1. Using a flow cytometry-based method, we noticed a moderate shift in the TCR clonotype use of CD8^+^ T cells. Although this effect is rather modest, Lai *et al.* observed a comparable response within the tumor-specific OT-I CD8^+^ T cell subset,^27^ while we assessed the endogenous bulk CD8^+^ T cell population. Using TCR sequencing, we found that CD8α ALN-1 empowers epitope spreading in the TDLN by increasing the frequency of multiple unique T cell clones in the TDLN. In accordance with our flow cytometry data, CD8α ALN-1 facilitated trafficking of different T cell clones from the TDLN to the TME and enables their oligoclonal expansion at the tumor site. While the results from these experiments are convincing, more pronounced epitope spreading could theoretically be expected in absence of OVA, which is a relatively immunogenic model antigen. How IL-1 exactly mediates these effects warrants further research and more insight in this process could be extremely valuable to improve effectivity of adjuvants focused at driving cellular immunity. A potential mechanism could be lowering of the antigen sensitivity threshold of CD8^+^ T cells by modulation of downstream TCR signaling, which has been observed for other proinflammatory cytokines, including type I IFN, IL-12 and the IL-1 superfamily member IL-18.^34 35^

Unlike human cDC1s, murine cDC1s express CD8α^36^ and represent an additional target for CD8α ALN-1. Indeed, we noticed upregulation of the activation markers CD40 and CD86 on TDLN cDC1s after treatment with CD8α ALN-1, which is in line with earlier reports.^15 37^ As can be expected from the critical role of cDC1s in the cancer-immunity cycle,^1^ basal proliferation of OT-I CD8^+^ T cells in response to TAA was strongly reduced in full-body Batf3^-/-^ mice, which lack a functional cDC1 compartment. While less T cells engaged in cell division, CD8α ALN-1 could still empower CD8^+^ T cells that initiated proliferation, independent of cDC1s. Effector differentiation of endogenous CD8^+^ T cells by CD8α ALN-1 was not influenced in absence of cDC1s, yet we did observe differences in T-bet and Eomes expression. Eomes^+^T-bet^dim^ CD8^+^ T cells were increased in Batf3^-/-^ mice after CD8α ALN-1 treatment, a phenotype described by Jia *et al.* to be functionally impaired and correlated with poor prognosis in acute myeloid leukemia.^38^ However, these results should be cautiously interpreted, as recent studies reported on the importance of T cell-intrinsic Batf3 expression during memory CD8^+^ T cell and T_H_9 CD4^+^ T cells development.^39 40^

Cancer vaccines are challenged by the vast amount of tumor mass the immune system must face. Vaccine-induced CD8^+^ T cells need to take on billions of cancer cells in immunosuppressive environments, which contributes to their limited efficacy.^12^ Results from clinical studies have shown that cancer vaccines can elicit measurable reactions, but these seldomly evolve into an objective response.^41^ We therefore sought to combine OT-I CD8^+^ T cell infusion with CD8α ALN-1 administration, as some studies have suggested that combination of ATCT and cancer vaccination could be a valuable strategy.^42 43^ While delivery of OT-I CD8^+^ T cells alone had no effect on tumor growth, additional administration of only three shots of CD8α ALN-1 prolonged tumor control, even causing complete responses in some mice. CD8α ALN-1 strongly shifted the phenotype of the infused T cells towards an effector phenotype, characterized by a CD44^+^CD62L^-^ profile and T-bet expression, in the TDLN and the tumor. These results are in accordance with data shown by Lee *et al.*, who demonstrated comparable effects with WT IL-1β.^18^ Of note, more pronounced effects on OT-I CD8^+^ T cell proliferation after treatment with CD8α ALN-1 could be observed in LLC-OVA compared with B16-OVA, which can potentially be explained by differences in OVA presentation, composition of the TME^44^ and lesion sizes at the time of T cell infusion. Revitalization of endogenous T cells could be essential for achieving optimal therapeutic efficiency of ATCT.^45^ Combination of OT-I CD8^+^ T cell transfer with CD8α ALN-1 treatment not only activated the transferred T cells, but also elicited comparable effects within the endogenous CD8^+^ T cell population. We measured a decrease in terminally dysfunctional (CD101^+^/CD38^+^) and an increase in stem cell-like (TCF-1^+^) endogenous CD8^+^ T cells in tumors of mice that received CD8α ALN-1, which has previously been described to be essential for durable antitumor responses to immunotherapy.^46 47^ The observed upregulation of PD-1 on OT-I CD8^+^ T cells following their activation by CD8α ALN-1 also provides a rationale to investigate whether additional combination treatment with anti-PD-1 or anti-PD-L1 monoclonal antibodies could further enhance therapeutic efficacy.

We went on to study the therapeutic effect of the combination between CD8α ALN-1 and CD13 AFR, which represents an AcTakine that delivers TNF to tumor endothelial cells by targeting the neovasculature marker aminopeptidase N (CD13).^31 48^ The rationale behind this combination is the dual effect that can potentially be achieved: (1) enhanced expansion and effector differentiation of antitumor CD8^+^ T cells; and (2) improved tumor trafficking as a consequence of tumor neovasculature activation. Earlier work demonstrated that CD13 AFR acts on HUVECs to drive ICAM-1 expression,^31^ which mediates TME infiltration of activated CD8^+^ T cells.^49^ Sequential delivery of CD8α ALN-1 and CD13 AFR induced rapid and complete tumor necrosis, characterized by conversion of the tumor mass into a scab that eventually disappeared and completely healed. Mice that received this treatment remained tumor-free for several weeks and were protected against a secondary challenge, which indicates the presence of immunological memory.

This effect strikingly mirrored the phenotype that results from combined intratumoral delivery of CD13 AFR and a CD13 IFN-γ-based AcTakine, which has been reported in earlier work by our group.^31^ The synergy between IFN-γ and TNF has been extensively documented and recent evidence showed that IFN-γ acts on endothelial cells to enhance TNF sensitivity, probably by upregulation of TNF-RI or apoptotic mediators, such as pro-caspase 8.^31 50^ We hypothesized that antitumor CD8^+^ T cells form a local source of IFN-γ upon their activation by CD8α ALN-1. In the TME, IFN-γ can subsequently lower the threshold for TNF activity, enabling full tumor eradication upon CD13 AFR delivery. Tumor clearance and absence of TAAs allow local CD8^+^ T cells to differentiate into memory cells, which safeguard the host against rechallenge. We measured more IFN-γ release by CD8^+^ T cells *ex vivo* and an increase in circulating IFN-γ after treatment with CD8α ALN-1. Moreover, IFN-γRI blockade prevented the synergy between CD8α ALN-1 and CD13 AFR.

The cytokine and chemokine analysis we performed showed that CD13 AFR treatment induces an elevation of IL-10 levels. IL-10 executes anti-inflammatory functions and has been described before to be upregulated by TNF, representing a negative feedback that controls excessive tissue inflammation.^51^ CD13 AFR alone also mediates expression of CCR and CXCR family chemokines, indicating that different innate and adaptive immune cells are being recruited to the TME. How these heterogeneous immune populations exactly influence the synergy between IL-1 and TNF activity in the tumor demands further research. Of note, only the sequential delivery of CD8α ALN-1 and CD13 AFR induced IL-2 and IL-17A release, suggesting the initiation of Treg and T_H_17 immunity.

While B16 and LLC are commonly used models in cancer research due to their robustness and fast growth, among other reasons, the simplicity of these models represents an important limitation of our study. In order to narrow the gap between bench and bedside, the clinical potential of CD8α ALN-1 as a therapeutic cancer vaccine adjuvant should therefore be further evaluated in orthotopically growing tumor models and patient-derived xenografts.

## Conclusion

Delivery of IL-1 activity to CD8^+^ T cells bypasses toxicities and unwanted side effects, while driving proliferation, activation and effector functionality of antitumor T cells. CD8α ALN-1 improves the efficacy of ATCT and synergizes with neovasculature-targeted TNF for full tumor eradication. The synergy between CD8α ALN-1 and CD13 AFR is mediated by IFN-γ, probably produced upon CD8^+^ T cell activation. This AcTakine/cytokine triumvirate acts as a powerful ‘nuke’ that mediates tumor destruction and holds promise for further clinical translation.

10.1136/jitc-2021-003293.supp2 Supplementary data

## Data Availability

Data are available in a public, open access repository. All data relevant to the study are included in the article or uploaded as online supplemental information.
