# Supplementary material for: Selective IL-1 activity on CD8+ T cells empowers antitumor immunity and synergizes with neovasculature-targeted TNF for full tumor eradication
Source: J Immunother Cancer. 2021 Nov 12;9(11):e003293. doi: 10.1136/jitc-2021-003293 (PMC8593706; doi:10.1136/jitc-2021-003293)
Supplement: Supplementary data [file jitc-2021-003293supp001.pdf]

Supplementary Materials for

Selective IL-1 activity on $CD8^+$ T cells empowers antitumor immunity and synergizes with neovasculature-targeted TNF for full tumor eradication

Bram Van Den Eeckhout, Leander Huyghe, Sandra Van Lint, Elianne Burg, Stéphane Plaisance, Frank Peelman, Anje Cauwels, Gilles Uzé, Niko Kley, Sarah Gerlo* and Jan Tavernier*

*Corresponding authors. Email: sarah.gerlo@ugent.be and jan.tavernier@vib-ugent.be

These Supplementary Materials include:

- Supplementary Figures and Table Legends
- Supplementary Figures 1 – 16
- Supplementary Table 1 – 2
- Supplementary Methods
- Supplementary References

Van Den Eeckhout B, *et al. J Immunother Cancer* 2021; 9:e003293. doi: 10.1136/jitc-2021-003293

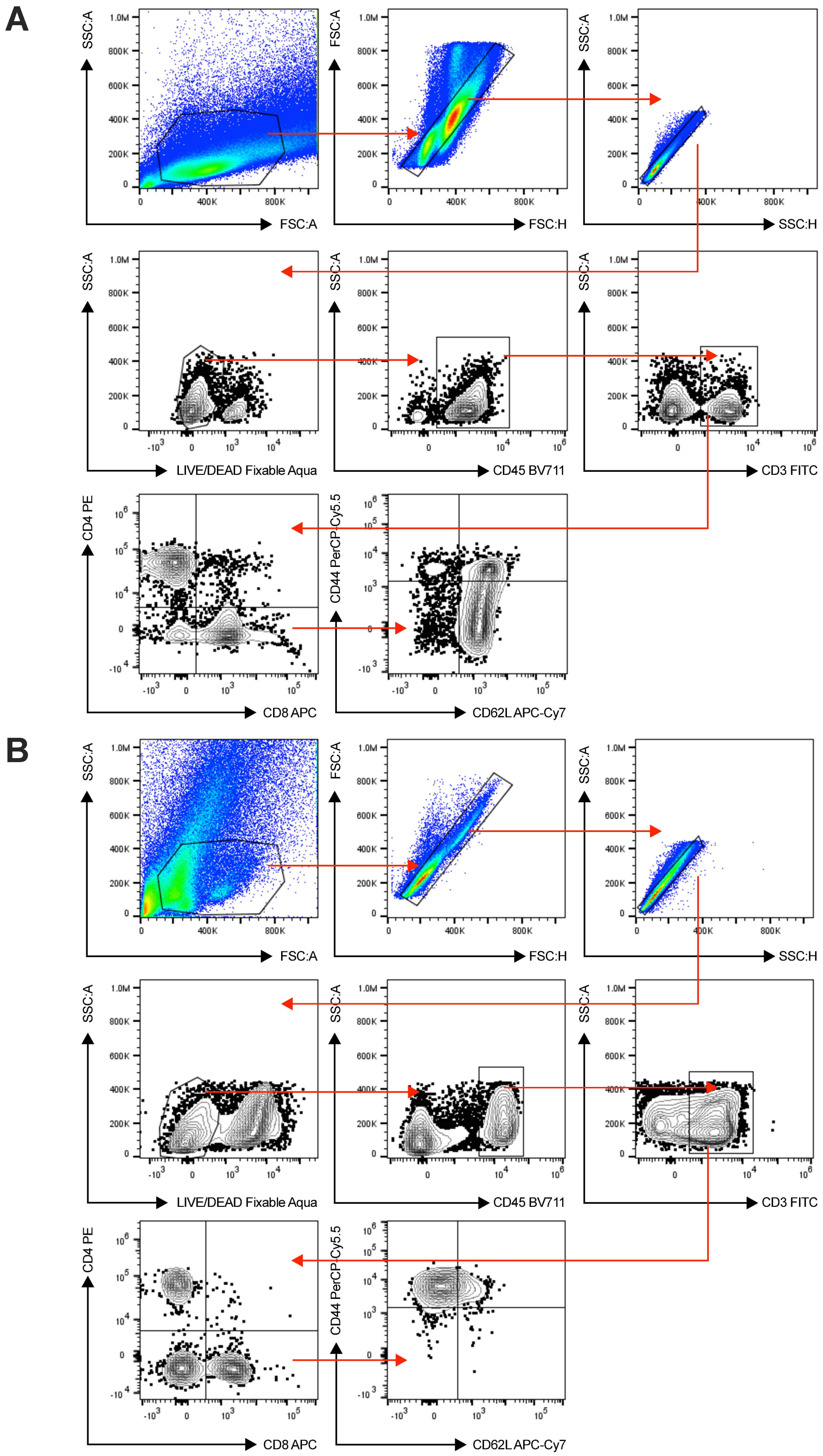
A
SSC:A
FSC:A
FSC:H
SSC:H
LIVE/DEAD Fixable Aqua
CD45 BV711
CD3 FITC
CD4 PE
CD8 APC
CD44 PerCP-Cy5.5
CD62L APC-Cy7
B

Supplementary Figure 1. Gating strategy for the detection of naive and effector CD8^{+} T cells in the TDLN (**A**) and tumor tissue (**B**) of LLC-OVA tumor-bearing mice via flow cytometry. Staining with LIVE/DEAD Fixable Aqua cell viability dye, CD45 BV711, CD3 FITC, CD4 PE, CD8 APC, CD44 PerCP-Cy5.5 and CD62L APC-Cy7. In the living (LIVE/DEAD^{-}) subset of single cells (based on FSC/SSC), we detected naive CD8^{+} T cells as CD45^{+}CD3^{+}CD4^{-}CD8^{+}CD44^{-}CD62L^{+} cells and effector CD8^{+} T cells as CD45^{+}CD3^{+}CD4^{-}CD8^{+}CD44^{+}CD62L^{-} cells.

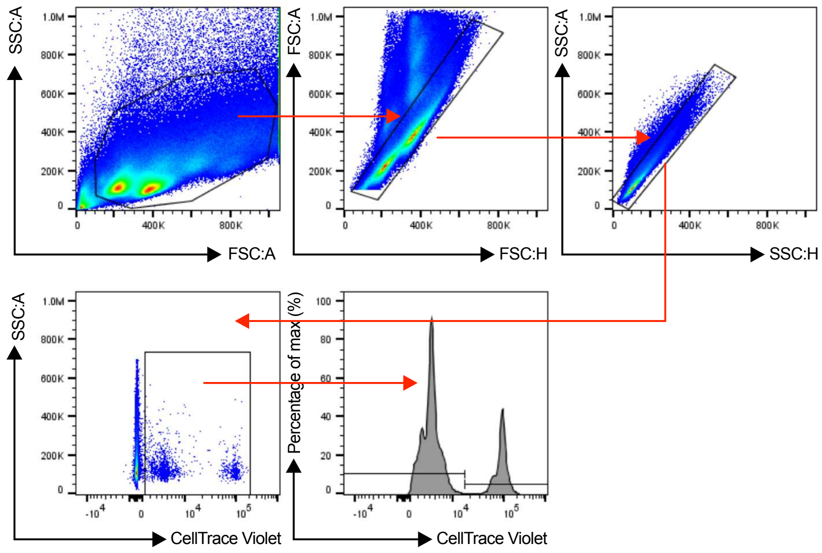

Supplementary Figure 2. Gating strategy for the detection of adoptively transferred target cells in the spleens of acceptor mice. Prior to transfer, cells were labeled with CTV in a high or low concentration and loaded with SIINFEKL (CTV^{high}) or left unloaded (CTV^{low}).

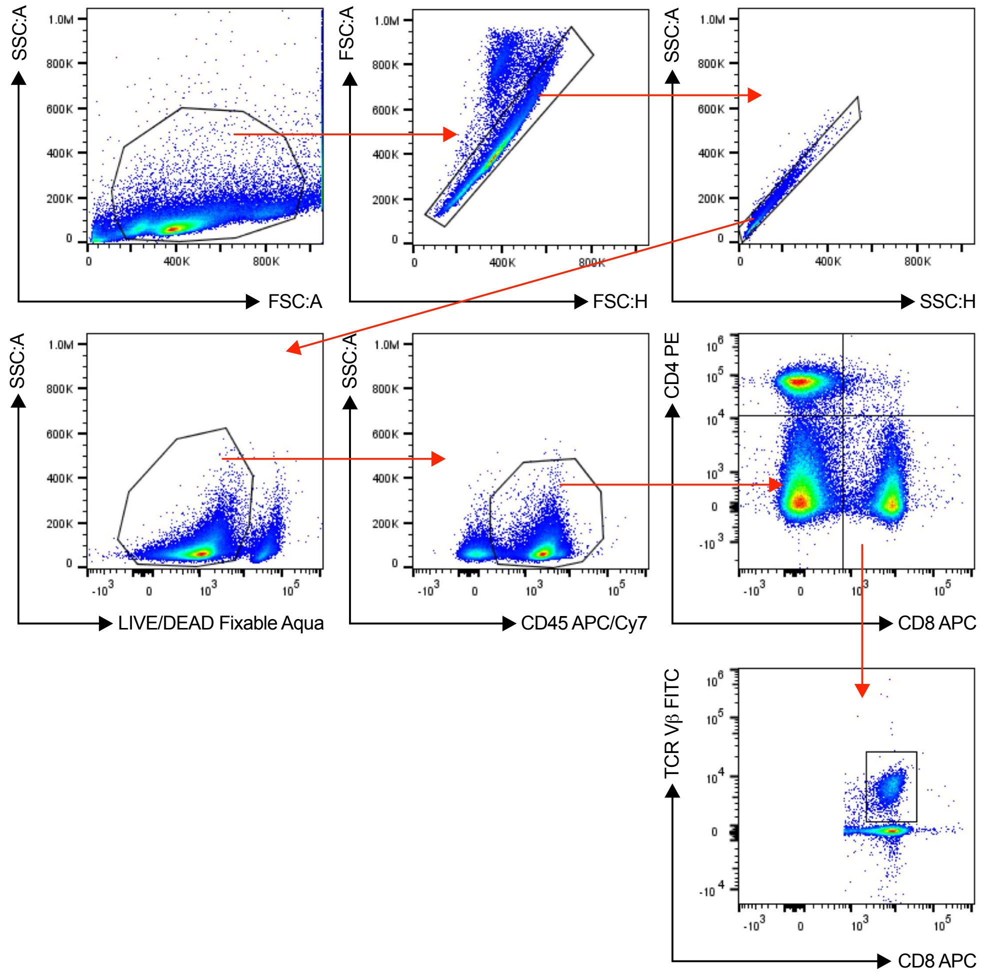

Supplementary Figure 3. Gating strategy for the detection of different TCR Vβ clonotypes in the TDLN of LLC-OVA tumor-bearing mice via flow cytometry. Staining with LIVE/DEAD Fixable Aqua cell viability dye, CD45 APC-Cy7, CD4 PE, CD8 APC and different TCR Vβ FITC antibodies. Clonotype frequencies were measured in the $CD45^+CD4^-CD8^+$ and $CD45^+CD4^+CD8^-$ subsets of living (LIVE/DEAD$^-$) single cells (based on FSC/SSC).

A

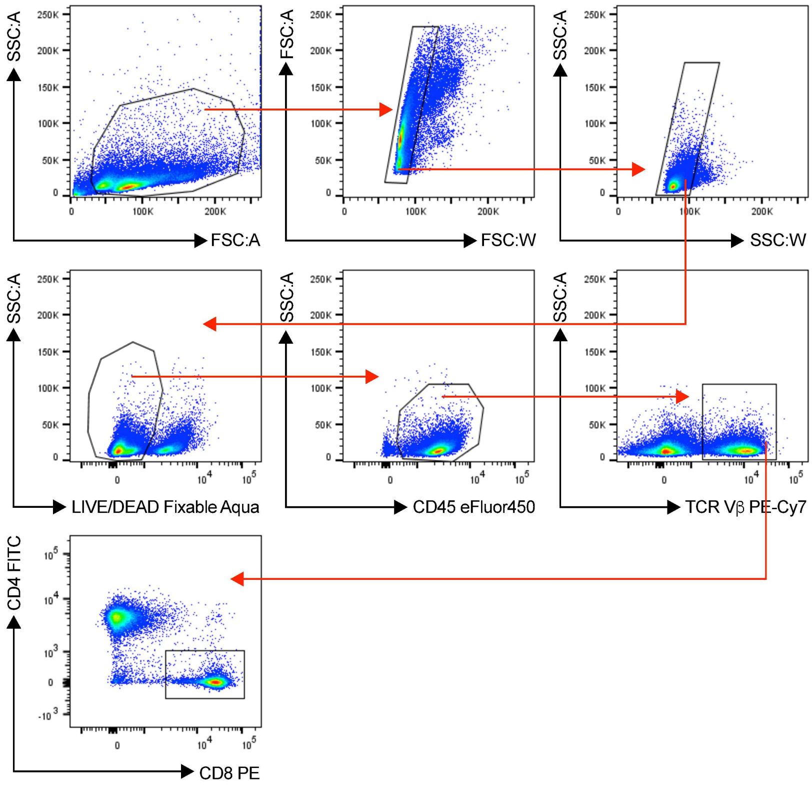
SSC:A
FSC:A
FSC:A
FSC:W
SSC:A
SSC:W
SSC:A
LIVE/DEAD Fixable Aqua
SSC:A
CD45 eFluor450
SSC:A
TCR Vβ PE-Cy7
CD4 FITC
CD8 PE

B

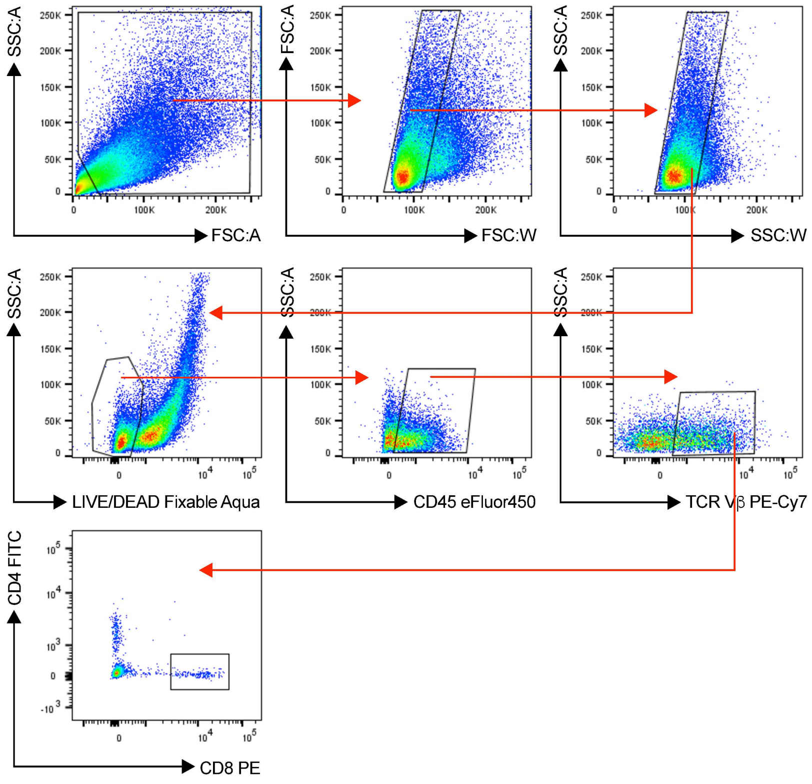
SSC:A
FSC:A
FSC:A
FSC:W
SSC:A
SSC:W
SSC:A
LIVE/DEAD Fixable Aqua
SSC:A
CD45 eFluor450
SSC:A
TCR Vβ PE-Cy7
CD4 FITC
CD8 PE

Supplementary Figure 4. Gating strategy for the sort of $CD8^+$ T cells from the TDLN (**A**) and tumor tissues (**B**) of LLC-OVA tumor-bearing mice. Staining with LIVE/DEAD Fixable Aqua cell viability dye, CD45 eFluor450, TCR Vβ PE-Cy7, CD4 FITC and CD8 PE. In the living ($LIVE/DEAD^-$) subset of single cells (based on FSC/SSC), we detected $CD8^+$ T cells as $CD45^+TCR\ V\beta^+CD4^-CD8^+$ cells.

Van Den Eeckhout B, *et al. J Immunother Cancer* 2021; 9:e003293. doi: 10.1136/jitc-2021-003293

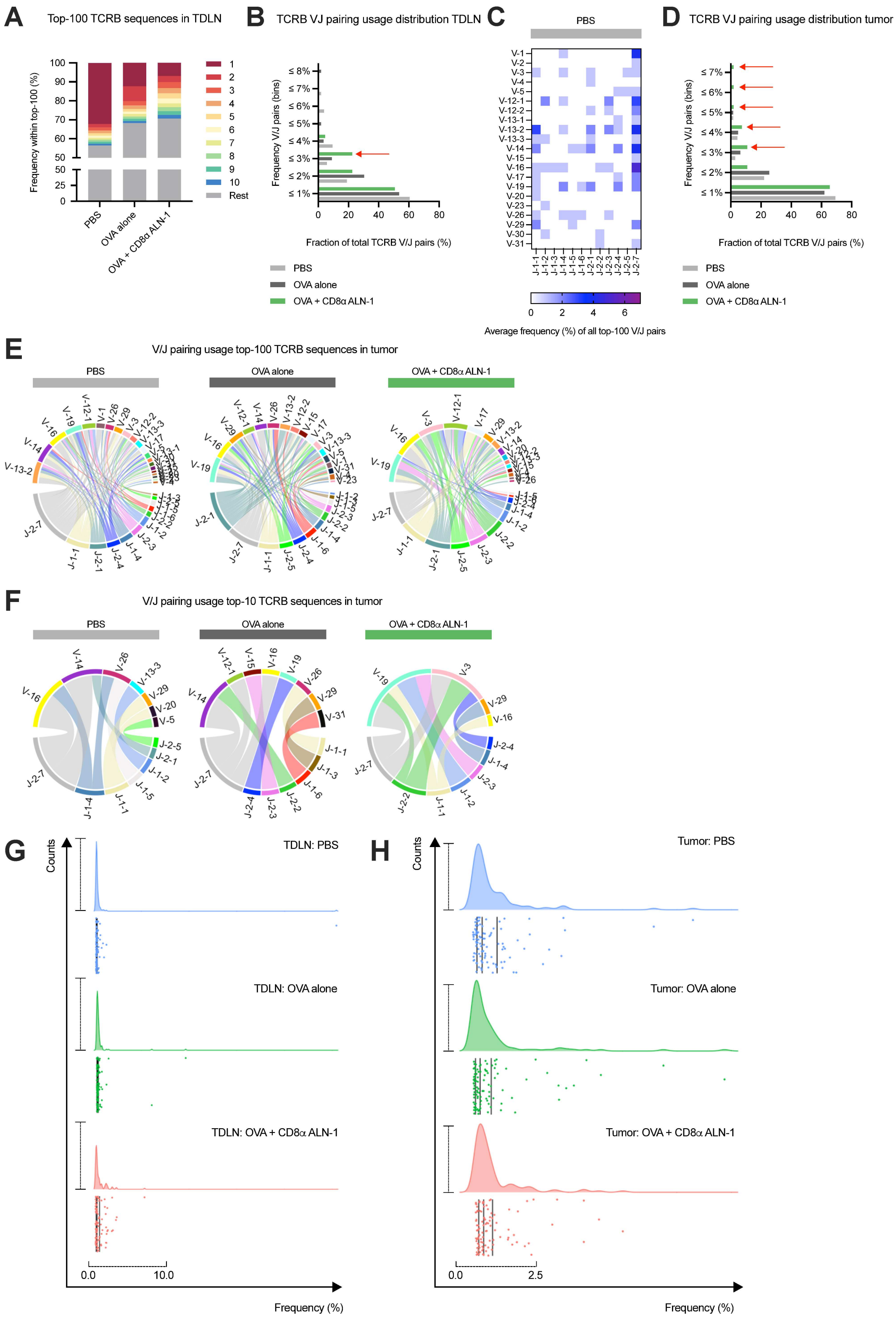
A
Top-100 TCRB sequences in TDLN
Frequency within top-100 (%)
PBS
OVA alone
OVA + CD8α ALN-1
Rest
B
TCRB VJ pairing usage distribution TDLN
Frequency V/J pairs (bins)
Fraction of total TCRB V/J pairs (%)
PBS
OVA alone
OVA + CD8α ALN-1
C
PBS
Average frequency (%) of all top-100 V/J pairs
D
TCRB VJ pairing usage distribution tumor
Frequency V/J pairs (bins)
Fraction of total TCRB V/J pairs (%)
E
V/J pairing usage top-100 TCRB sequences in tumor
F
V/J pairing usage top-10 TCRB sequences in tumor
G
TDLN: PBS
TDLN: OVA alone
TDLN: OVA + CD8α ALN-1
Counts
Frequency (%)
H
Tumor: PBS
Tumor: OVA alone
Tumor: OVA + CD8α ALN-1
Counts
Frequency (%)

7

Supplementary Figure 5. (**A**) Frequency of the top-10 most abundant TCRB sequences (stacked bars in color) within the top-100 most abundant TDLN TCRB sequences. The same color does not necessarily represent the same clonotype in the different bars. Data from individual mice were pooled per group. (**B**) **and** (**D**) Frequency distribution of V/J pairing usage of the top-100 most abundant TCRB sequences in the TDLN (**B**) and tumor (**D**). (**C**) V/J pairing usage within the top-100 most abundant TDLN TCRB sequences for mice treated with PBS. (**E**) **and** (**F**) V/J pairing usage within the top-100 (**E**) and top-10 (**F**) most abundant tumor TCRB sequences. (**G**) **and** (**H**) Frequency distribution of the top-100 most abundant TCRB sequences in TDLN (**G**) and tumor (**H**). Histograms (*top*) give an overview of the frequency distribution per group with dotted lines indicating the scale for both axes. Points *(bottom)* represent the individual clones that comprise the top-100 TCRB sequences. Black vertical lines represent Q1, Q2 (= median) and Q3.

Van Den Eeckhout B, *et al. J Immunother Cancer* 2021; 9:e003293. doi: 10.1136/jitc-2021-003293

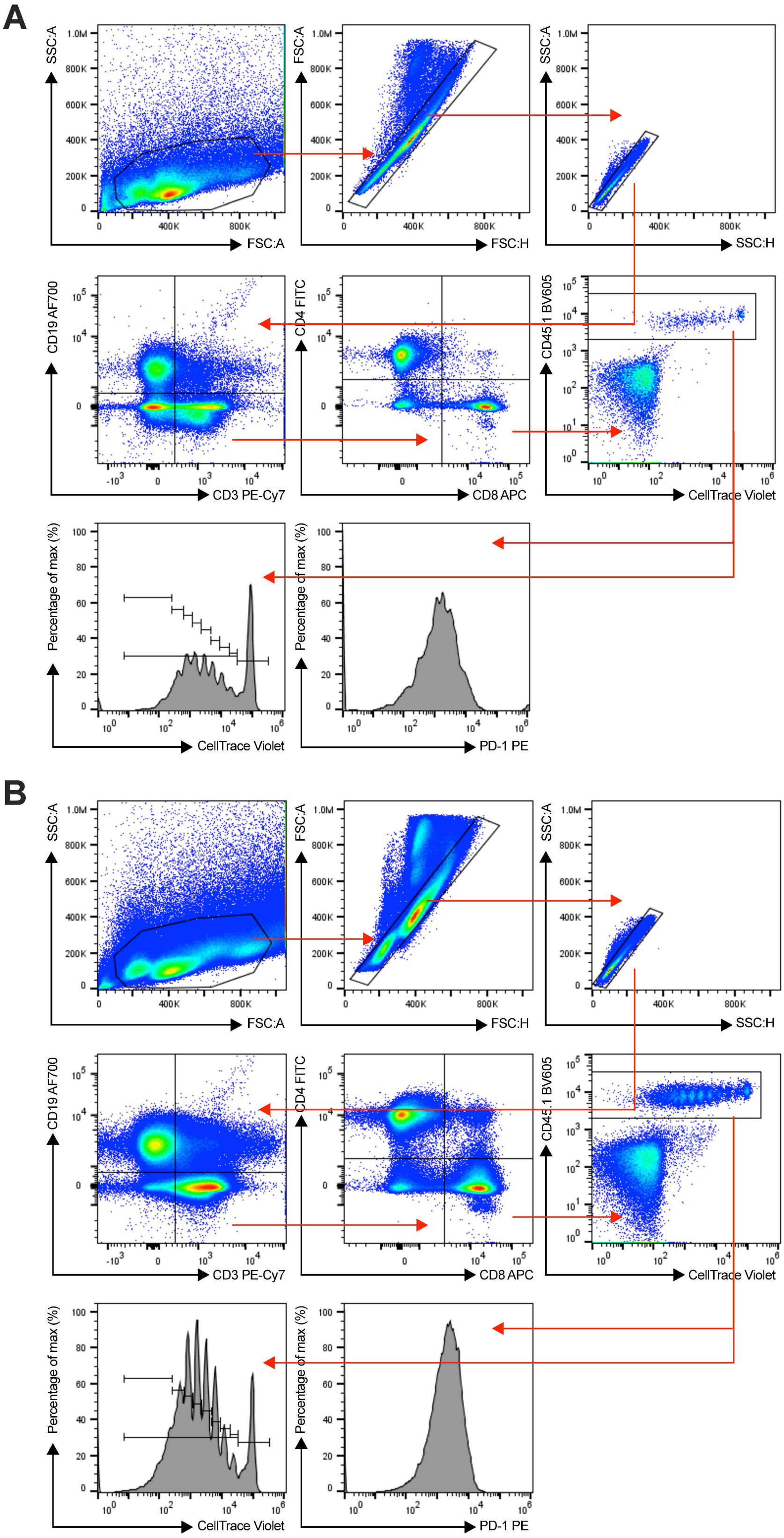
A
SSC:A
FSC:A
FSC:A
FSC:H
SSC:A
SSC:H
CD19 AF700
CD3 PE-Cy7
CD4 FITC
CD8 APC
CD45.1 BV605
CellTrace Violet
Percentage of max (%)
CellTrace Violet
Percentage of max (%)
PD-1 PE
B
SSC:A
FSC:A
FSC:A
FSC:H
SSC:A
SSC:H
CD19 AF700
CD3 PE-Cy7
CD4 FITC
CD8 APC
CD45.1 BV605
CellTrace Violet
Percentage of max (%)
CellTrace Violet
Percentage of max (%)
PD-1 PE

Supplementary Figure 6. Gating strategy for the detection of OT-I CD8$^+$ T cell proliferation and PD-1 upregulation in the TDLNs of B16- (**A**) and LLC-OVA (**B**) tumor-bearing mice via flow cytometry. Staining with CD19 AF700, CD3 PE-Cy7, CD4 FITC, CD8 APC and CD45.1 BV605. OT-I CD8$^+$ T cells were detected as CD19$^-$CD3$^+$CD4$^-$CD8$^+$CD45.1$^+$CTV^{labeled} cells within the single cell population (based on FSC/SSC).

Van Den Eeckhout B, *et al. J Immunother Cancer* 2021; 9:e003293. doi: 10.1136/jitc-2021-003293

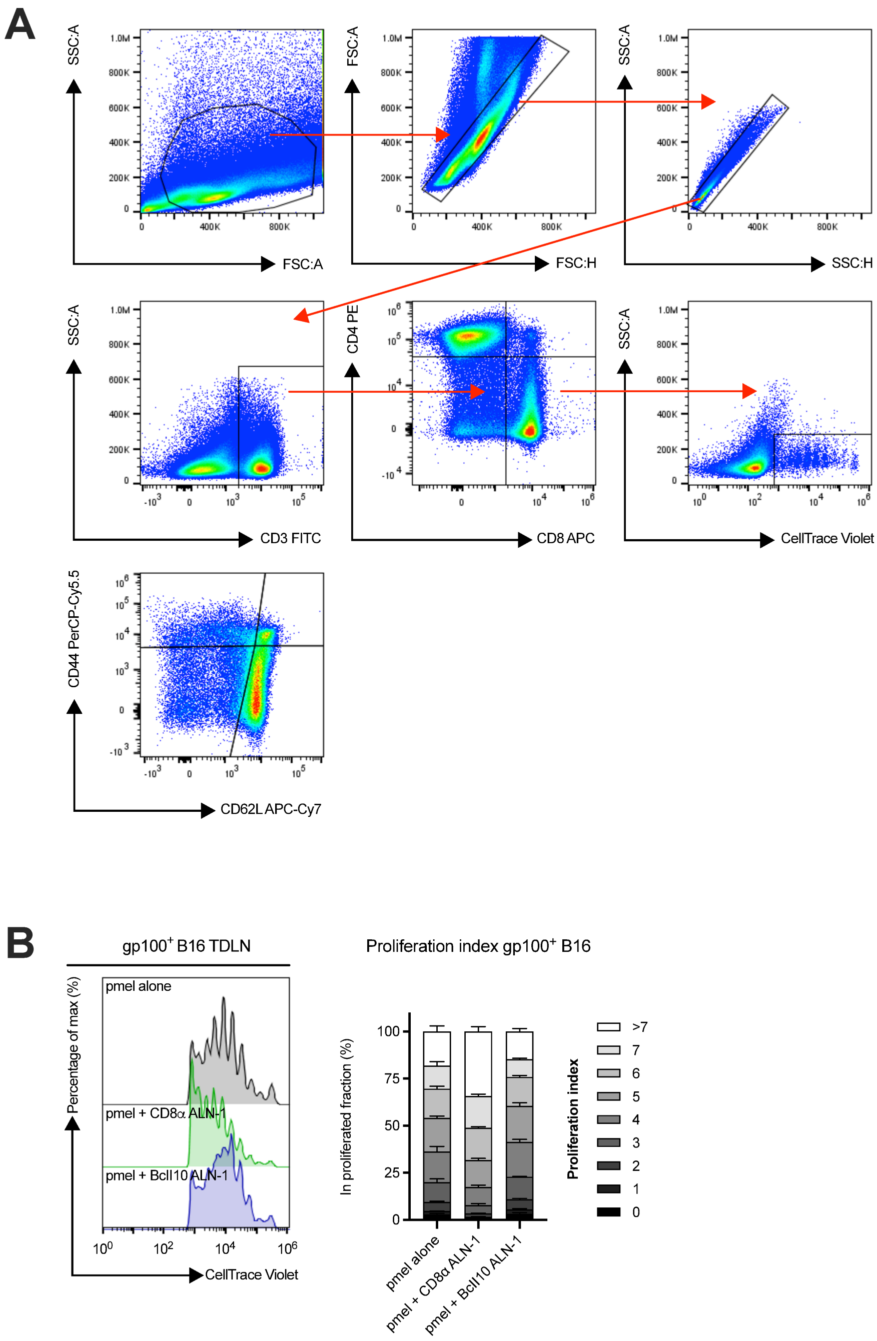

Supplementary Figure 7. (**A**) Gating strategy for the detection of pmel CD8$^+$ T cell proliferation in the TDLNs of B16 gp100$^+$ tumor-bearing mice via flow cytometry. Staining with CD3 FITC, CD4 PE and CD8 APC. pmel CD8$^+$ T cells were detected as CD3$^+$CD4$^-$CD8$^+$CTV^{labeled} cells within the single cell population (based on FSC/SSC). (**B**) Stacked

histograms summarizing the proliferation index of pmel $CD8^+$ T cell proliferation in B16 $gp100^+$ tumor-bearing mice. Individual stacks represent the mean percentages of proliferating pmel $CD8^+$ T cells in certain stages of cell division ± s.e.m. of an experiment with $n = 6$ mice/group. Representative flow cytometry histograms display pmel $CD8^+$ T cell proliferation.

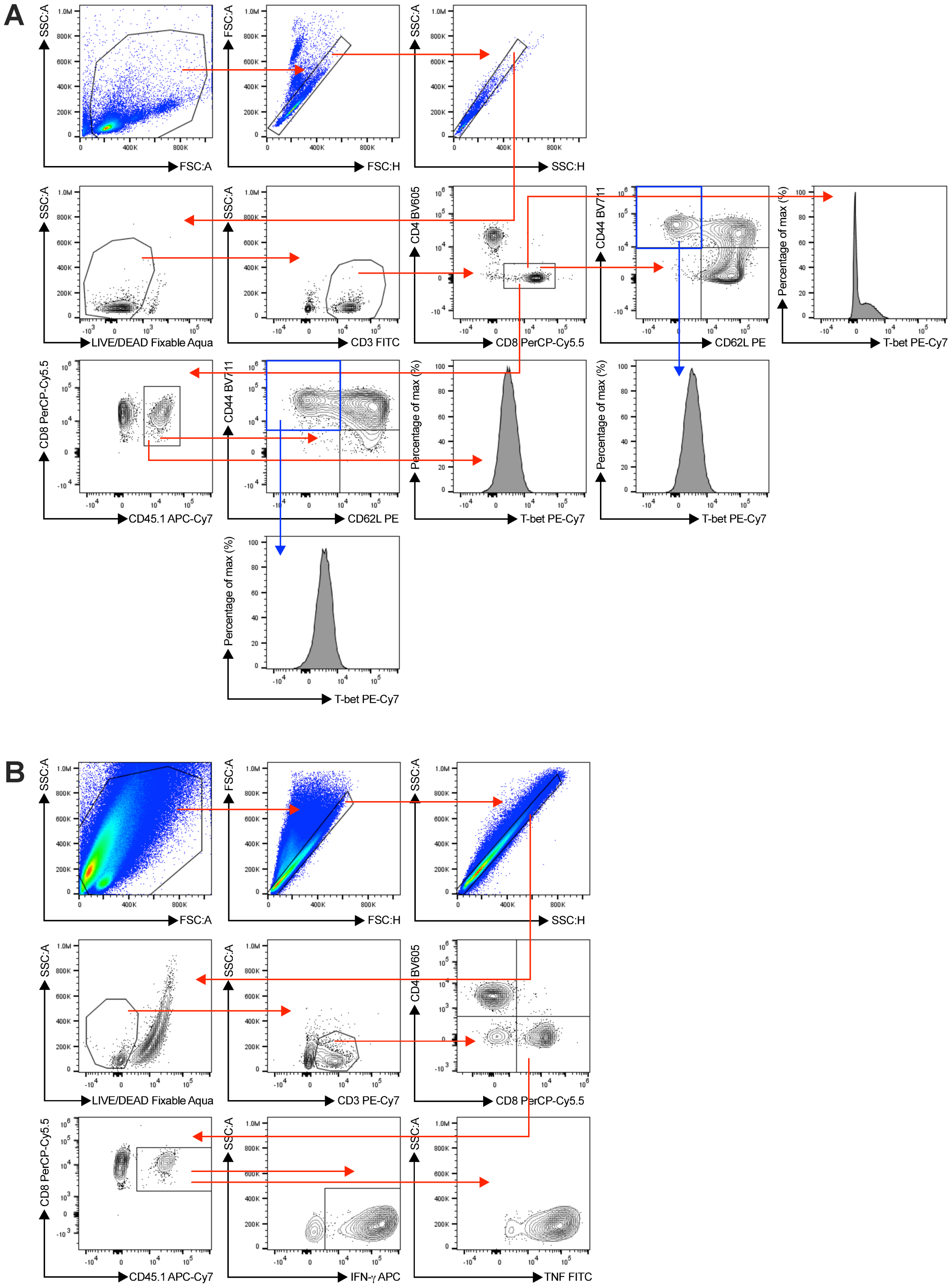

Supplementary Figure 8. (**A**) Gating strategy for the detection of naive and effector (OT-I) CD8⁺ T cells in the TDLN and tumor tissue of LLC-OVA tumor-bearing mice. Staining with

LIVE/DEAD Fixable Aqua cell viability dye, CD3 FITC, CD4 BV605, CD8 PerCP-Cy5.5, CD45.1 APC-Cy7, CD44 BV711, CD62L PE and T-bet PE-Cy7. Naive and effector (OT-I) $CD8^+$ T cells were identified within the living ($LIVE/DEAD^-$) single cell (based on FSC/SSC) population as $CD3^+CD4^-CD8^+(CD45.1^+)CD44^-CD62L^+$ cells and $CD3^+CD4^-CD8^+(CD45.1^+)CD44^+CD62L^-$ cells, respectively. (**B**) Gating strategy for the detection of cytokine release following *ex vivo* restimulation of murine splenocytes with SIINFEKL peptide. Staining with LIVE/DEAD Fixable Aqua cell viability dye, CD3 PE-Cy7, CD4 BV605, CD8 PerCP-Cy5.5, CD45.1 APC-Cy7, IFN-γ APC and TNF FITC. We detected IFN-γ and TNF release in the $CD3^+CD4^-CD8^+(CD45.1^+)$ cells within the population of living ($LIVE/DEAD^-$) single cells (based on FSC/SSC).

Van Den Eeckhout B, *et al. J Immunother Cancer* 2021; 9:e003293. doi: 10.1136/jitc-2021-003293

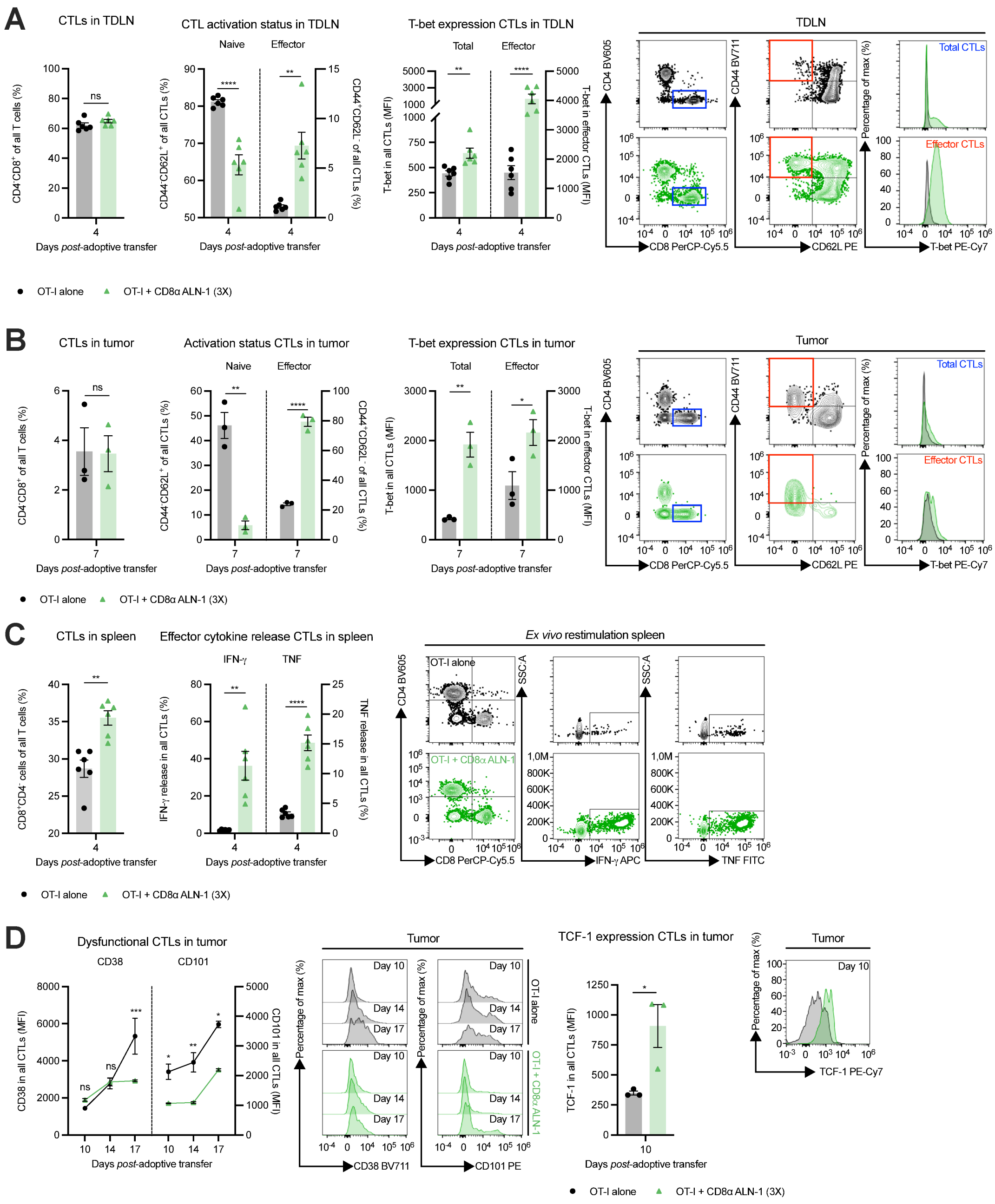

Supplementary Figure 9. (**A**) **and** (**B**) Frequencies of CD8$^+$ T cells within the total T cell population, naive (CD44$^-$CD62L$^+$) and effector (CD44$^+$CD62L$^-$) phenotypes within the total CD8$^+$ T cell population and T-bet expression within the total and effector CD8$^+$ T cell population in TDLN (**A**) and tumor tissue (**B**) with representative flow cytometry dot plots. (**C**) Frequencies of CD8$^+$ T cells within the total T cell population and release of IFN-γ and

TNF by CD8$^+$ T cells following *ex vivo* restimulation of splenocytes with SIINFEKL peptide with representative flow cytometry dot plots. (**D**) Expression of CD101, CD38 and TCF-1 on CD8$^+$ T cells in tumor with representative flow cytometry histograms. Bars and data points represent the mean ± s.e.m. of an experiment with $n = 6$ mice/group (for TDLN and spleen samples) or $n = 3$ mice/group (for tumor samples, as two mice were pooled together). ****, $p < 0.0001$; ***, $p < 0.001$; **, $p < 0.01$; *, $p < 0.05$; ns, $p \geq 0.05$ by unpaired Student's t-test (**A – D**) or two-way ANOVA with Sidak's multiple comparison's test (**D**).

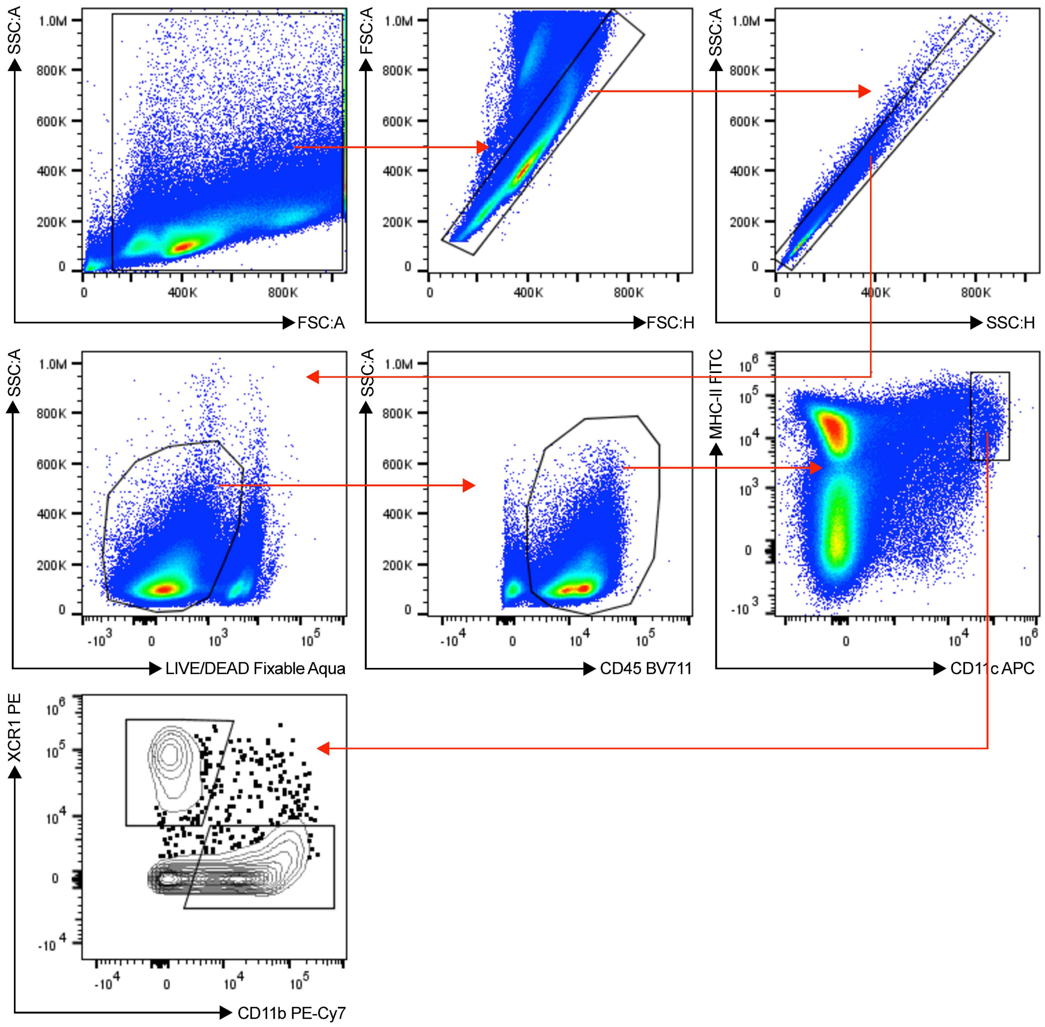

Supplementary Figure 10. Gating strategy for the detection of cDC1s and cDC2 in the TDLNs of LLC-OVA tumor-bearing mice via flow cytometry. Staining with LIVE/DEAD Fixable Aqua cell viability dye, CD45 BV711, MHC-II FITC, CD11c APC, XCR1 PE and CD11b PE-Cy7. Within the subset of living (LIVE/DEAD$^-$) single cells (based on FSC/SSC), we detected cDC1s as CD45$^+$MHC-II$^+$CD11c$^+$XCR1$^+$CD11b$^-$ cells and cDC2 as CD45$^+$MHC-II$^+$CD11c$^+$XCR1$^-$CD11b$^+$ cells.

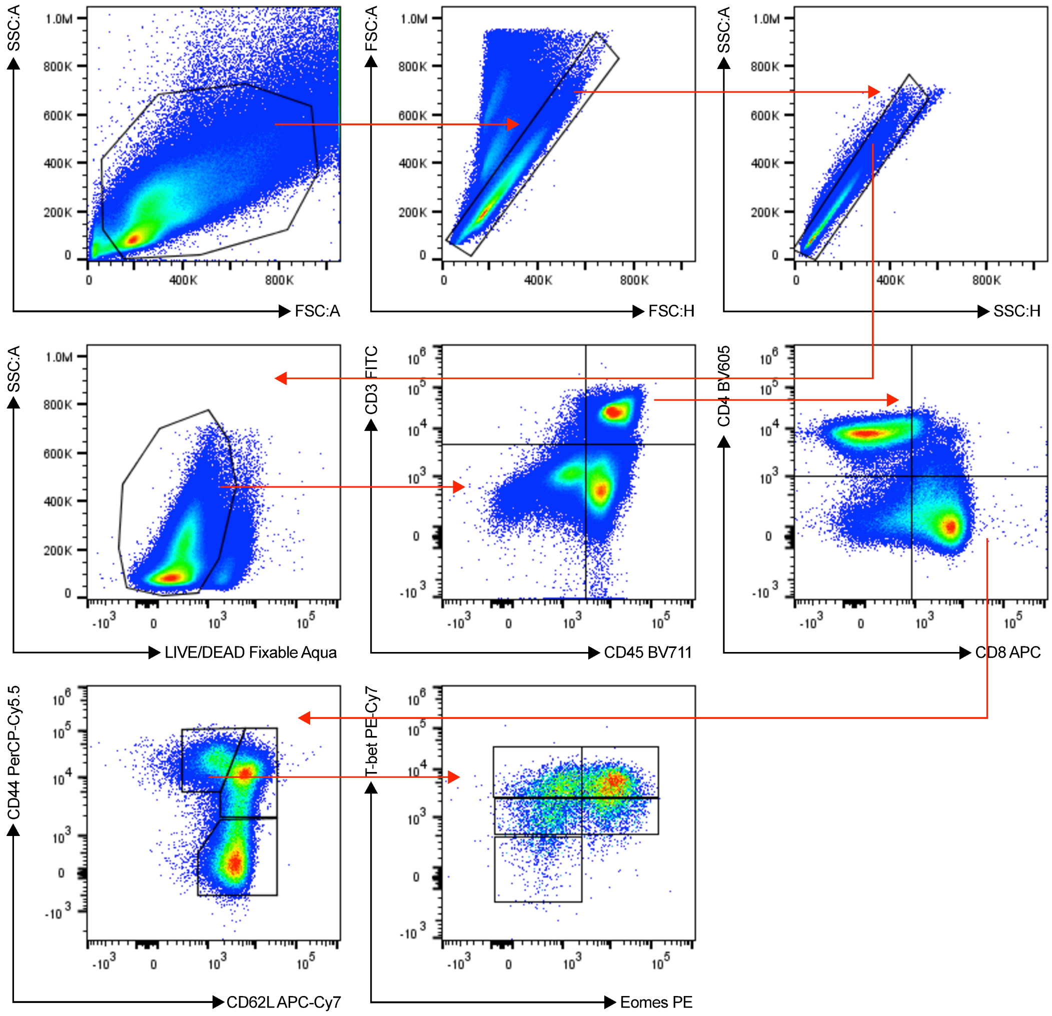

Supplementary Figure 11. Gating strategy for the detection of naive and effector CD8$^+$ T cells in the TDLNs of WT or full-body Batf3$^{-/-}$ C57BL/6 mice. Staining with LIVE/DEAD

Van Den Eeckhout B, *et al. J Immunother Cancer* 2021; 9:e003293. doi: 10.1136/jitc-2021-003293

Fixable Aqua cell viability dye, CD3 FITC, CD45 BV711, CD4 BV605, CD8 APC, CD44 PerCP-Cy5.5, CD62L APC-Cy7, T-bet PE-Cy7 and Eomes PE. In the living (LIVE/DEAD$^-$) subset of single cells (based on FSC/SSC), we detected naive CD8$^+$ T cells as CD45$^+$CD3$^+$CD4$^-$CD8$^+$CD44$^-$CD62L$^+$ cells and effector CD8$^+$ T cells as CD45$^+$CD3$^+$CD4$^-$CD8$^+$CD44$^+$CD62L$^-$ cells.

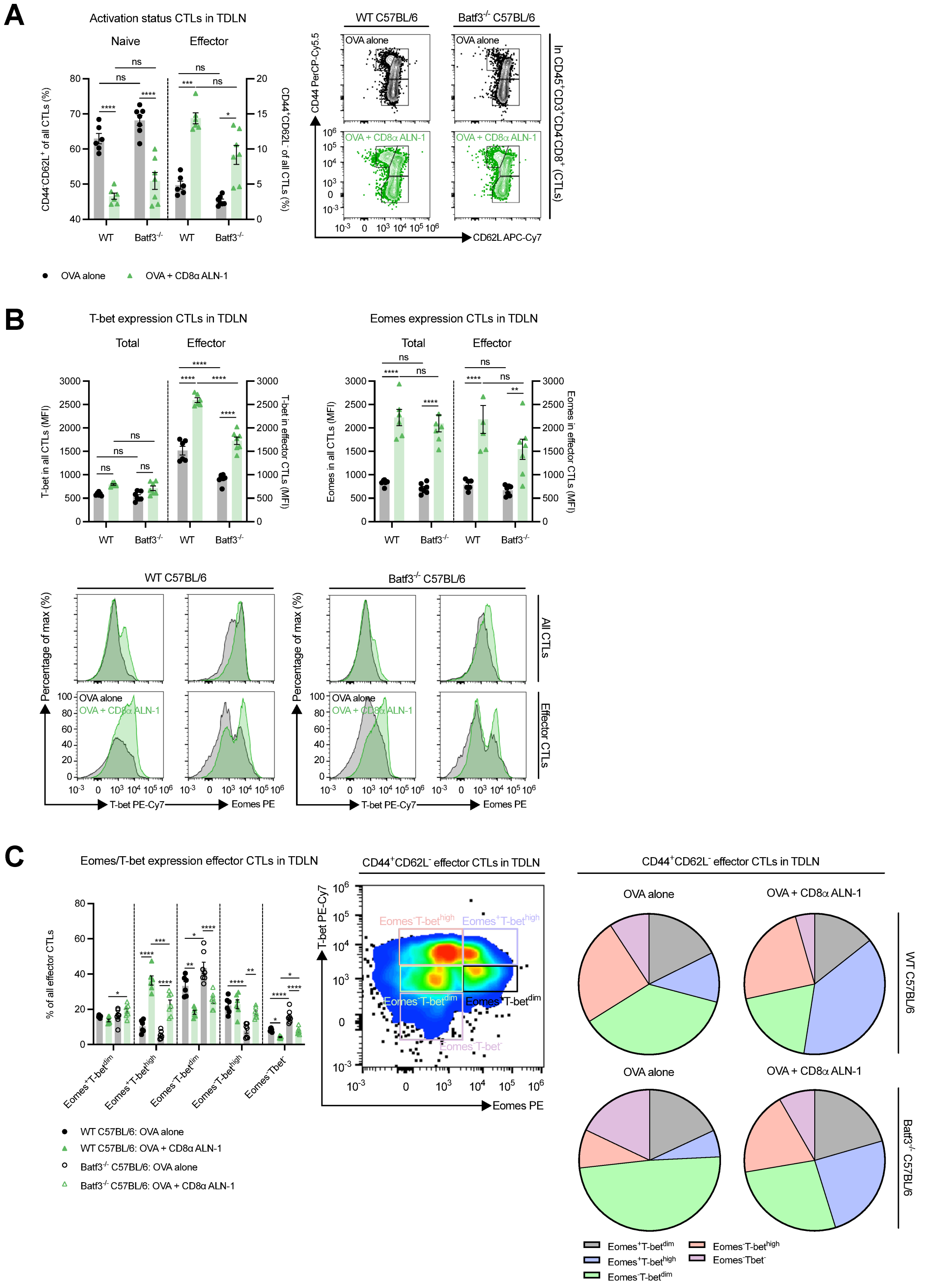

Supplementary Figure 12. (**A**) Relative amounts of naive (CD44⁻CD62L⁺) and effector (CD44⁺CD62L⁻) CD8⁺ T cells within the total CTL population in TDLNs of WT or full-body

Batf3$^{-/-}$ LLC-OVA tumor-bearing mice (*left*) with representative flow cytometry dot plots (*right*). (**B**) Expression (MFI) of T-bet and Eomes in the total or effector CD8^{+} T cell population in TDLNs of WT or full-body Batf3$^{-/-}$ LLC-OVA tumor-bearing mice (*top*) with representative flow cytometry histograms (*bottom*). (**C**) Relative amounts of different T-bet/Eomes-expressing effector CD8^{+} T cell populations within TDLNs of WT or full-body Batf3$^{-/-}$ LLC-OVA tumor-bearing mice (*left*) according to the gating strategy displayed in the representative flow cytometry dot plot (*middle*). Fractions of the different T-bet/Eomes-expressing effector CD8^{+} T cell population represented as parts-of-whole (*right*). Bars represent the mean ± s.e.m. of an experiment with $n = 6$ mice/group. ****, $p < 0.0001$; ***, $p < 0.001$; **, $p < 0.01$; *, $p < 0.05$; ns, $p \geq 0.05$ by one-way ANOVA with Tukey's multiple comparisons test.

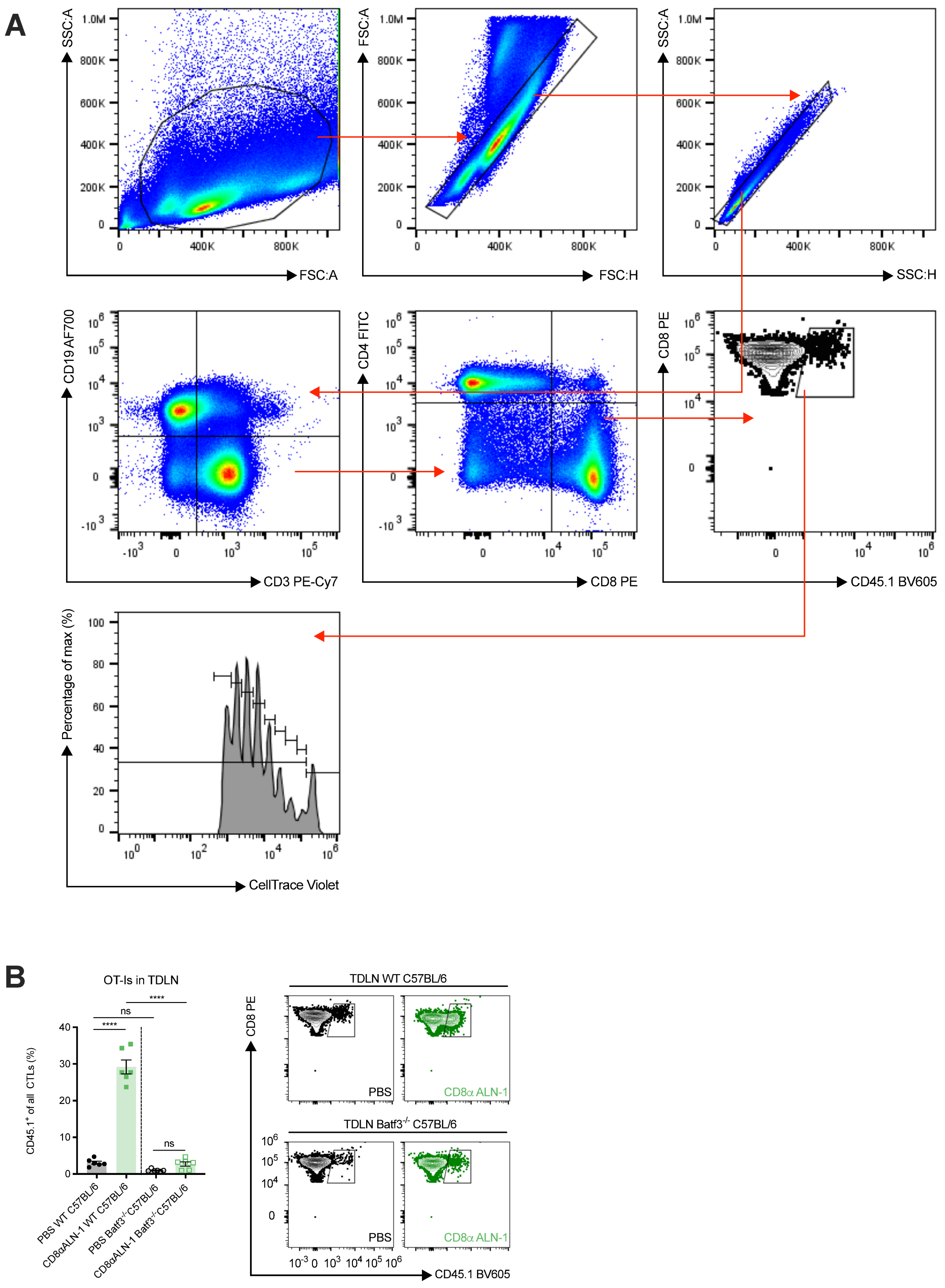

Supplementary Figure 13. (**A**) Gating strategy for the detection of OT-I CD8$^+$ T cell proliferation in the TDLNs of WT or full-body Batf3$^{-/-}$ C57BL/6 mice. Staining with CD19

AF700, CD3 PE-Cy7, CD4 FITC, CD8 PE and CD45.1 BV605. OT-I CD8$^+$ T cells were detected as CD19$^-$CD3$^+$CD4$^-$CD8$^+$CD45.1$^+$ cells within the single cell population (based on FSC/SSC) and proliferation was measured as CTV dilution. (**B**) Relative amounts of OT-I CD8$^+$ T cells within the total CD8$^+$ T cell population in TDLNs of WT and full-body Batf3$^{-/-}$ LLC-OVA tumor-bearing acceptor mice (*left*) with representative flow cytometry dot plots (*right*). Bars represent the mean ± s.e.m. of an experiment with n = 6 mice/group. ****, p < 0.0001; ns, $p \geq 0.05$ by one-way ANOVA with Tukey's multiple comparisons test.

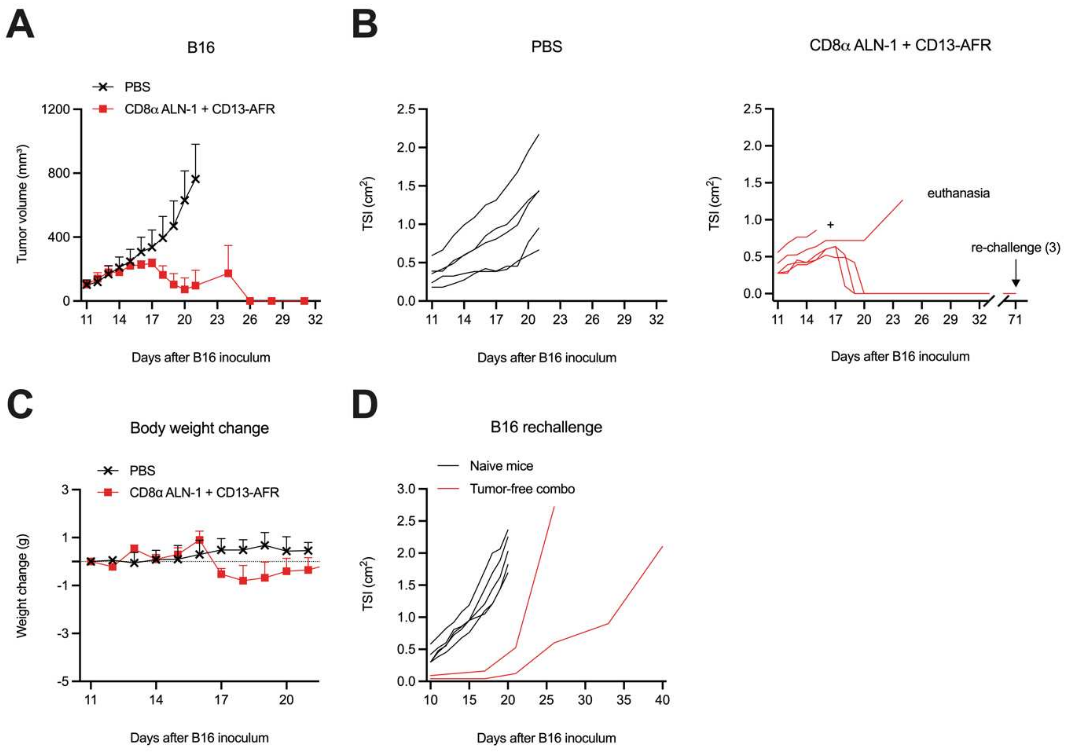

Supplementary Figure 14. Mice were inoculated with B16 (0.6x10^6 cells) and treatment was initiated after 10 days of s.c. tumor growth. From D10 – D13, mice received an i.p. treatment with CD8α ALN-1 (10 µg) combined with additional i.p. treatments with CD13 AFR (50 µg) from D14 – D16. Control mice received i.p. treatments with PBS from D10 – D16. (**A**) B16 tumor growth over time. Data points represent the mean ± s.e.m. of one experiment with n = 5

mice/group. (**B**) B16 tumor growth over time for the individual treatment conditions. Individual mice are shown. (**C**) Change in body weight over time. Data points represent the mean ± s.e.m. of one experiment with $n = 5$ mice/group. (**D**) B16 tumor growth over time after s.c. rechallenge of mice previously treated with CD8α ALN-1 + CD13 AFR. Naive mice were used as control animals in this experiment. Individual mice are shown.

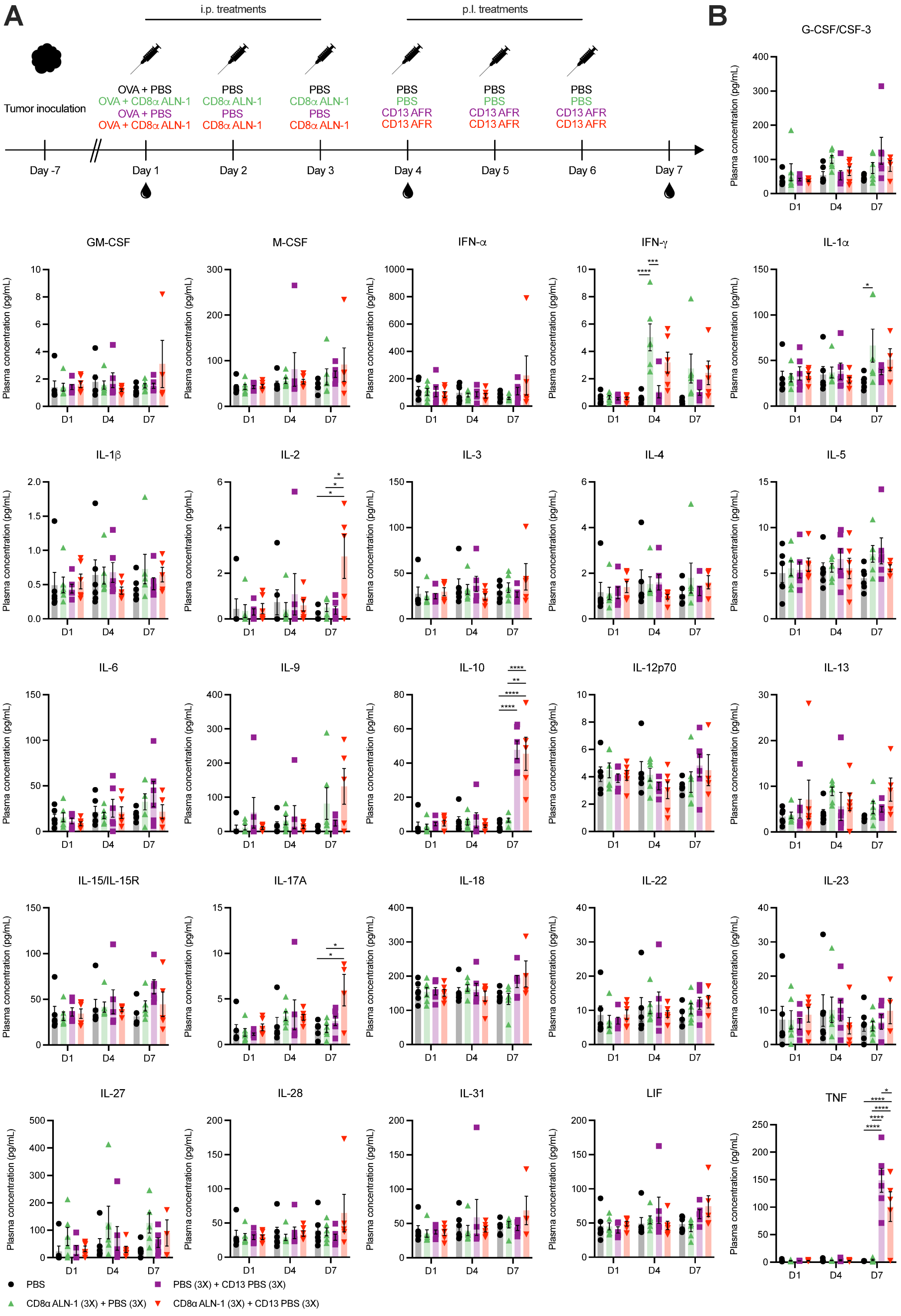
A
i.p. treatments
p.I. treatments
Tumor inoculation
OVA + PBS
OVA + CD8α ALN-1
OVA + PBS
OVA + CD8α ALN-1
PBS
CD8α ALN-1
PBS
CD8α ALN-1
PBS
PBS
CD13 AFR
CD13 AFR
Day -7
Day 1
Day 2
Day 3
Day 4
Day 5
Day 6
Day 7
B
G-CSF/CSF-3
GM-CSF
M-CSF
IFN-α
IFN-γ
IL-1α
IL-1β
IL-2
IL-3
IL-4
IL-5
IL-6
IL-9
IL-10
IL-12p70
IL-13
IL-15/IL-15R
IL-17A
IL-18
IL-22
IL-23
IL-27
IL-28
IL-31
LIF
TNF
Plasma concentration (pg/mL)
D1
D4
D7
PBS
PBS (3X) + CD13 PBS (3X)
CD8α ALN-1 (3X) + PBS (3X)
CD8α ALN-1 (3X) + CD13 PBS (3X)

Supplementary Figure 15. (**A**) Schematic representation of the experiment. LLC-OVA tumors were grown s.c. for seven days, after which mice received i.p. treatments with OVA (100 µg) in combination with PBS or CD8α ALN-1 (10 µg) every 24h for three consecutive days, followed by p.l. treatments with PBS or CD13 AFR (50 µg) every 24h for three consecutive days. Tail vein blood was sampled at the start of treatment (day 1), the fourth day of treatment (day 4) or one day after the last treatment (day 7). (**B**) Plasma cytokine concentrations following different treatments over time. Bars represents the mean ± s.e.m. of an experiment with $n = 6$ mice/group. ****, $p > 0.0001$; ***, $p > 0.001$; **, $p > 0.01$; *, $p > 0.05$; ns, $p \geq 0.05$ by one-way ANOVA with Tukey's multiple comparisons test.

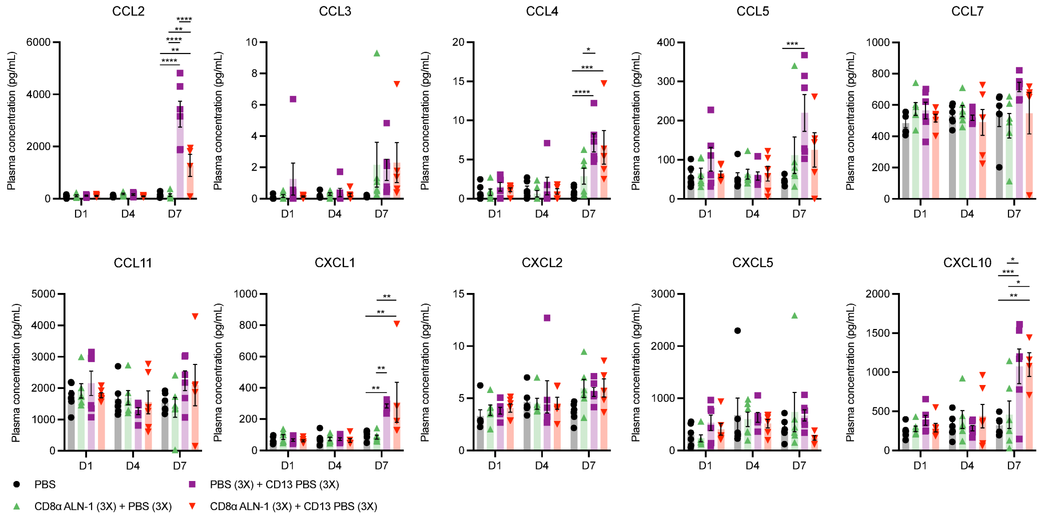

Supplementary Figure 16. Plasma chemokine concentrations following different treatments over time. Bars represents the mean ± s.e.m. of an experiment with $n = 6$ mice/group. ****, $p > 0.0001$; ***, $p > 0.001$; **, $p > 0.01$; *, $p > 0.05$; ns, $p \geq 0.05$ by one-way ANOVA with Tukey's multiple comparisons test.

Van Den Eeckhout B, *et al. J Immunother Cancer* 2021; 9:e003293. doi: 10.1136/jitc-2021-003293

Target and fluorochrome	Dilution	Clone	Category number	Supplier
CD16/32	100	clone 93	14-0161-82	Thermo Fisher Scientific
CD19 AF700	500	clone 145-2C11	552774	BD Biosciences
CD4 FITC	250	clone RM4-5	100510	BioLegend
CD8 APC	250	clone 53-6.7	553035	BD Biosciences
CD8 PE	250	clone 53-6.7	12-0081-82	Thermo Fisher Scientific
CD45.1 BV605	250	clone A20	110737	BioLegend
PD-1 PE	100	clone J43	12-9985-82	Thermo Fisher Scientific
LIVE/DEAD Fixable Aqua cell viability dye	1000		L34957	Thermo Fisher Scientific
CD45 BV711	500	clone 30-F11	103147	BioLegend
CD3 FITC	250	clone 17A2	555274	BD Biosciences
CD4 PE	250	clone RM4-5	553048	BD Biosciences
CD4 BV605	250	clone RM4-5	100547	BioLegend
CD44 PerCP-Cy5.5	100	clone IM7	103032	BioLegend
CD62L APC-Cy7	100	clone MEL-14	104428	BioLegend
T-bet PE-Cy7	100	clone 4B10	644823	BioLegend
Eomes PE	100	clone Dan11mag	12-4875-82	Thermo Fisher Scientific
MHC-II FITC	250	clone M5/114.15.2	11-5321-82	Thermo Fisher Scientific
CD11c APC	250	clone N418	117310	BioLegend
XCR1 PE	100	clone ZET	148204	BioLegend
CD11b PE-Cy7	250	clone M1/70	101215	BioLegend
CD40 Pacific Blue	100	clone 3/23	124626	BioLegend
CD86 APC-Cy7	100	clone GL-1	105030	BioLegend
CD45 APC-Cy7	500	clone 30-F11	103116	BioLegend
TCR Vb FITC (15 clonotypes)			557004	BD Biosciences
CD45 eFluor450	500	clone 30-F11	48-0451-82	Thermo Fisher Scientific
TCR Vb PE-Cy7	250	clone KT11	125916	BioLegend
CD8 PerCP-Cy5.5	250	clone 53-6.7	100733	BioLegend
CD44 BV711	100	clone IM7	103057	BioLegend
CD62L PE	100	clone MEL-14	104407	BioLegend
CD45.1 APC-Cy7	250	clone A20	560579	BD Biosciences
CD38 BV711	100	clone 90/CD38	740697	BD Biosciences
CD101 PE	100	clone Moushi101	12-1011-82	Thermo Fisher Scientific
TCF-1 PE-Cy7	100	clone C63D9	90511	Cell Signaling Technology
IFN-g APC	100	clone XMG1.2	562019	BD Biosciences
TNF FITC	100	clone MP6-XT22	506303	BioLegend

Supplementary Table 1. Complete list of antibodies and dyes used in this manuscript.

TCR clonotype	OVA alone group	OVA + CD8α ALN-1 group	Change compared with OVA alone
Vβ2	4,928333333	5,046666667	0,118333333
Vβ3	2,77	2,83	0,06
Vβ4	3,675	3,746666667	0,071666667
Vβ5.1/2	13,06666667	11,93333333	-1,133333333
Vβ6	6,658333333	6,406666667	-0,251666667
Vβ7	5,843333333	5,828333333	-0,015
Vβ8.1/2	15,61666667	15,33333333	-0,283333333
Vβ8.3	6,46	6,528333333	0,068333333
Vβ9	1,883333333	2,331666667	0,448333333
Vβ10b	4,938333333	5,143333333	0,205
Vβ11	5,085	6,043333333	0,958333333
Vβ12	2,541666667	2,518333333	-0,023333333
Vβ13	3,648333333	3,952	0,303666667
Vβ14	2,548333333	2,853333333	0,305
Vβ17b	0,82	0,505	-0,315
Others	19,51666667	18,99966667	-0,517

Supplementary Table 2. Average frequencies and change in average frequencies of $CD8^+$ TCR Vβ clonotypes. Mice were treated with OVA alone or OVA + CD8α ALN-1 according to the treatment scheme presented in Fig. 4A (*n* = 6 mice/group).

Van Den Eeckhout B, *et al. J Immunother Cancer* 2021; 9:e003293. doi: 10.1136/jitc-2021-003293

Supplementary Methods

Recombinant protein design, production and purification

Site-directed mutagenesis was used to introduce the Q148G and Y86F mutation in the coding sequence of human IL-1β and murine TNF, respectively[1,2]. Generation and selection of sdAbs was performed by the VIB Protein Service Facility[1–3]. Proteins were constructed in an in-house developed vector, produced in mammalian FreeStyleTM 293-F cells (R79007, Thermo Fisher Scientific) and purified from supernatants as described elsewhere[1]. Purity was evaluated by SDS-PAGE and Instant Blue (EP ISB1L, Expedeon) staining of the protein gel.

Cell lines and culture conditions

B16- and LLC-OVA cells (Damya Laoui, VIB-VUB) were cultured in DMEM (41966-052, Thermo Fisher Scientific) or RPMI-1640 (61870-044, Thermo Fisher Scientific) + 10% fetal bovine serum (FBS) (10270-106, Thermo Fisher Scientific). All cell lines were grown at 37°C in a humidified atmosphere containing 5% CO_2 and tested negative for mycoplasma (Venor™GeM Mycoplasma Detection Kit, PCR-based, MP0025, Sigma-Aldrich). We did not perform full authentication of the cell lines.

Mice

Female C57BL/6 mice were purchased from Charles River Laboratories. OT-I TCR transgenic CD45.1 Rag2$^{-/-}$ C57BL/6 mice (Bart Lambrecht, VIB-UGent) and full-body Batf3$^{-/-}$ C57BL/6 mice (Karine Breckpot, VUB) were bred in our facility. All mice were housed under pathogen-free conditions in IVCs and temperature-controlled environments with a 12h day/12h night cycle and received food and water *ad libitum*. FELASA guidelines were followed in all experiments and approval was obtained by the Ethical Committee of the Faculty of Medicine and Health Sciences (UGent) (ECD 17/80k, ECD 18/127k and ECD 16/07). Mice were

randomly allocated to treatments and investigators were not blinded during data collection and analysis.

Formulae

Tumor volumes (mm^3) were calculated as: [longest perpendicular side (mm) x shortest perpendicular side (mm) x shortest perpendicular side (mm)]/2. The percentage of SIINFEKL-specific cytolytic activity was calculated as: 100−[100 × (%CTV^{high} treated mice/%CTV^{low} treated mice)/(%CTV^{high} PBS-treated mice/%CTV^{low} PBS-treated mice)].

TCR sequencing, data processing and analysis (detail)

Briefly, RNA was reverse transcribed into cDNA with TCR-specific RT primers. Double-stranded cDNA was generated via second-strand synthesis, after which cDNA was end-repaired and A-tailed. Every original cDNA molecule was assigned a unique molecular identifier (UMI) by ligation of a 12-base random adaptor sequence. Target enrichment was performed via PCR with one primer against the constant region and another primer complementary to the adapter. The library was ultimately amplified via a second PCR, which also introduced platform-specific adaptor sequences and additional sample indices. Samples were then pooled and sequenced in two Illumina MiSeq v2 500 runs. The read data was merged and analyzed using the mouse "Immune Repertoire Analysis" workflow of the CLC Genomics Workbench (GWB) v21 (https://digitalinsights.qiagen.com/)[4]. The analysis was done using default parameters, except for "min UMI group size", which was set to 1. The obtained clonotype results were used to produce count tables and figures.

Statistical analyses and data presentation (detail)

Data sets were compared by unpaired Student's *t*-testing (two-tailed) for the statistical analysis of differences between two groups or ANOVA (one- or two-way) with Tukey's or Sidak's multiple comparisons test for the statistical analysis of differences between more than two

groups. Statistical analysis of differences between Kaplan–Meier survival curve was performed by log-rank testing.

Supplementary References

1. Van Den Eeckhout, B. *et al.* Specific targeting of IL-1β activity to CD8+ T cells allows for safe use as a vaccine adjuvant. *npj Vaccines* **5**, 1–17 (2020).

2. Huyghe, L. *et al.* Safe eradication of large established tumors using neovasculature-targeted tumor necrosis factor-based therapies. *EMBO Mol. Med.* **12**, 1–15 (2020).

3. Garcin, G., Paul, F., Staufenbiel, M., Bordat, Y. & Van der Heyden, J. High efficiency cell-specific targeting of cytokine activity. *Nat. Commun* **5**, 1–9 (2014).

4. Bolotin, D. A. *et al.* MiXCR: software for comprehensive adaptive immunity profiling. *Nature Methods* vol. 12 380–381 (2015).
