# Supplementary material for: Selective IL-1 activity on CD8+ T cells empowers antitumor immunity and synergizes with neovasculature-targeted TNF for full tumor eradication
Source: J Immunother Cancer. 2021 Nov 12;9(11):e003293. doi: 10.1136/jitc-2021-003293 (PMC8593706; doi:10.1136/jitc-2021-003293)
Supplement: Supplementary data [file jitc-2021-003293supp002.pdf]

Selective IL-1 activity on CD8⁺ T cells empowers antitumor immunity and synergizes with neovasculature-targeted TNF for full tumor eradication

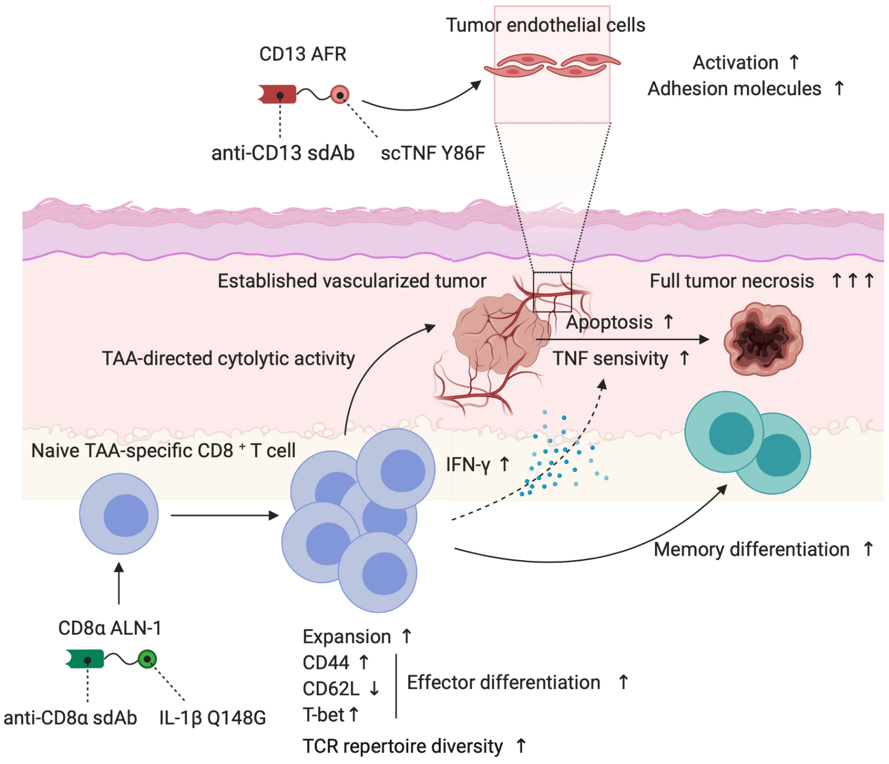

Authors
Bram Van Den Eeckhout, Leander Huyghe, Sandra Van Lint, Elianne Burg, Stéphane Plaisance, Frank Peelman, Anje Cauwels, Gilles Uzé, Niko Kley, Sarah Gerlo and Jan Tavernier

Correspondence
sarah.gerlo@ugent.be
jan.tavernier@vib-ugent.be

In Brief
AcTakines represent engineered cytokines that deliver cytokine activity to selected cell types only, as such evading damaging toxicities and unwanted off-target side effects. Here, an AcTakine that targets IL-1β activity to CD8⁺ T cells safely and efficiently drives antitumor cellular immunity. Combination with a neovasculature-targeted TNF AcTakine induces full tumor necrosis and establishes a protective immunological memory.
